# *LILRB1* and *LILRB2* genomics and transcriptomics in macaque and baboon species: polymorphism, diversification, and extensive alternative splicing

**DOI:** 10.3389/fimmu.2025.1706720

**Published:** 2026-01-09

**Authors:** Natasja G. de Groot, Nanine de Groot, Corrine M. C. Heijmans, Annemiek J. M. de Vos-Rouweler, Marit K. H. van der Wiel, Jesse Bruijnesteijn

**Affiliations:** Comparative Genetics and Refinement, Biomedical Primate Research Centre, Rijswijk, Netherlands

**Keywords:** alternative splicing, evolution, human, leucocyte immunoglobulin-like receptor (LILR), leucocyte receptor complex (LRC), long-tailed macaque, non-human primates (NHP), rhesus macaque

## Abstract

**Introduction:**

Inhibitory receptors play a pivotal role in fine-tuning immune responses. The leukocyte receptor complex (LRC) encodes multiple receptor families, including the leukocyte immunoglobulin-like receptor (LILR) family, which next to activating receptors involves several inhibitory receptors. The LILRB1 and LILRB2 receptors are considered immune checkpoint inhibitors, which may interact with MHC class I molecules, and are expressed mainly on monocytes, B- and T-cells.

**Method:**

In this study, we characterized *LILRB1* and *LILRB2* at the genomic and transcriptomic level in three Old World monkey species, namely rhesus and long-tailed macaques and Hamadryas baboon, using SMRT sequencing on PacBio platforms.

**Result and discussion:**

We describe 71 *LILRB1* and 58 *LILRB2* alleles in the two macaque species, of which only one allele was previously published. In contrast, less polymorphism is observed in the Hamadryas baboon, with only six *LILRB1* and seven *LILRB2* alleles characterized. Phylogenetic analysis, including known human data, revealed extensive diversification of the *LILRB1* and *LILRB2* in macaques, with allelic variation clustering into nine and twelve distinct lineages, respectively. This contrasts with the more conserved repertoires observed in humans and Hamadryas baboons. Compared with our experience analyzing *MHC* and *KIR* transcriptome data, the *LILRB1* and *LILRB2* transcriptomes were dominated by alternatively spliced isoforms. Alternative 3’ splice sites near exons 10 and 15 and/or skipping of exon 15, were encountered for most *LILRB1* alleles. In *LILRB2*, the deletion of exon 9 is the most prominent event, next to deletion of exon 10 and the use of alternative 3’ splice sites near exons 10 and 15. The exons that encode the extracellular domains remain largely intact, suggesting that alternative splicing predominantly affects the stem region and the signaling capacity of the LILRB1 and LILRB2 receptors.

## Introduction

The leukocyte immunoglobulin-like receptor (LILR) gene family is located on chromosome 19 within the leucocyte receptor complex (LRC), adjacent to the killer-cell immunoglobulin-like (KIR) region (1). The *LILR* gene cluster is organized into a centromeric and telomeric part separated by a highly conserved central region (1, 2). In humans, the *LILR* family comprises eleven functional genes, which encode for five activating (LILRA) and five inhibitory receptors (LILRB), and a soluble receptor, LILRA3 (3). In addition, two pseudogenes are present in this region. LILR receptors are involved in an array of functions, such as maintaining immune tolerance, inflammation, hematopoietic differentiation, and neural processes (3).

The *LILR* gene system appears to be an old entity, predating the emergence and radiation of the mammalian lineage roughly 100 million years ago (4, 5). In addition to humans, this system has been extensively studied in mice, where it is referred to as the paired immunoglobulin-like receptor (PIR) (6–8). In non-human primates (NHP), however, only limited data are available on *LILR* gene characterization. One of the better studied species is the chimpanzee (*Pan troglodytes*), which shared a common ancestor with humans approximately 5 to 6 million years ago. Nine different genes have been identified in chimpanzees, four of which are orthologous to human *LILR* genes, including *LILRB1* and *LILRB2* (9). A study in the rhesus macaque (*Macaca mulatta*, *Mamu*), which shared a common ancestor with humans approximately 25 to 33 million years ago (10, 11), highlighted that this species expresses various LILR receptors with similar structural features to their human counterparts (12). Previously, we published a thorough comparative analysis of the genomic *LILR* region across humans and different NHP species using available reference genome assemblies. This study demonstrated that the region has a highly conserved organization and revealed the presence of orthologs of the human *LILRB1* and *LILRB2* genes in apes, as well as in Old and New World monkey species (5).

The human *LILRB1* and *LILRB2* gene products, also known as LIR-1/ILT2/MIR-7/CD85j and LIR-2/ILT4/MIR-10/CD85d, respectively, each comprise of four Ig-like domains (D1-D4), stabilized by conserved disulfide bonds. Both proteins also contain a transmembrane region (TM) and a cytoplasmic tail (Cyt) that carries three to four immunoreceptor tyrosine-based inhibitory motifs (ITIM) (13). These ITIMs can recruit Src homology region 2 domain-containing phosphatase-1 (SHP-1), which is essential for inhibitory downstream signaling (14–16). The extracellular Ig-like domains can recognize human leucocyte antigen (HLA) class I molecules through the simultaneous interaction of domains D1 and D2 with the highly conserved α3 domain of HLA class I and the conserved β2-microglobulin (β2m) subunit, respectively (17). *LILRB2* gene products can also interact with β2m-free HLA molecules (18). Similarly, in mice, *PIR* gene products are shown to interact with major histocompatibility complex (MHC) class I molecules (6–8). Beyond HLA interaction, LILRB1 may engage with human cytomegalovirus HLA class I homolog (UL18) (14, 19), dengue virus (20), the damage-associated molecular pattern protein S100A9 (21), and plasmodium falciparum-expressed RIFIN proteins (22). LILRB2, on the other hand, can bind to CD1d (19), β-amyloid (23), and RIFIN (24). For example, the interaction between LILRB2 and β-amyloid may enhance the development of Alzheimer’s disease (AD) (23) and has been explored as a potential therapeutic target for treating AD (25, 26). Collectively, the diverse ligand binding profiles of LILRB1 and LILRB2 underlie their broad immunomodulatory functions.

Macaque species, such as the rhesus and long-tailed macaque (*Macaca fascicularis*, *Mafa*), are widely applied as model in translational research to study various aspects of human infectious diseases, including AIDS, COVID-19, Malaria and tuberculosis (27–30) as well as neurodegenerative diseases like AD and Parkinson’s disease (31, 32). Furthermore, naturally occurring cancers in macaques may provide valuable models for the development of human cancer immunotherapies (33). The interpretation of findings from such studies would benefit from detailed knowledge of the immune system of the model species. Given the roles of LILRB1 and LILRB2 as immune checkpoint receptors and as important ligands for HLA class I molecules, a comprehensive characterization of these genes in species applied in pre-clinical research is essential. Here, we present an in-depth characterization of *LILRB1* and *LILRB2* gene polymorphism at both the genomic and transcriptomic levels in rhesus (n=78) and long-tailed macaques (n=70). A smaller cohort of Hamadryas baboons (*Papio hamadryas*, *Paha*, n=29), was included for comparison. Like macaques, baboons are Old World monkeys (OWM), but they are native to Africa. They are also widely applied as models in various types of biomedical studies (34–36). For the analysis, we employed Single-Molecule Real-Time (SMRT) sequencing on Pacific Bioscience’s (PacBio) platforms. Previously, this method has been successfully applied by our team to characterize *KIR* transcriptional profiles in humans, chimpanzees, rhesus and long-tailed macaque families, as well as to investigate *MHC* allelic variation in macaques (37–41). In addition to uncovering extensive, previously unrecognized polymorphisms, we observed broad diversification of *LILRB1* and *LILRB2* alleles in rhesus and long-tailed macaques. Hamadryas baboons, however, exhibited a more conserved repertoire, similar to that observed in humans. *LILRB1* and *LILRB2* transcripts also display alternative splicing across all three non-human primate species, primarily affecting the exons encoding the stem and the signaling domains.

## Materials and methods

### Sample collection

The AAALAC-accredited facilities of the Biomedical Primate Research Centre (BPRC) house self-sustaining, pedigreed breeding colonies of rhesus and long-tailed macaques. These colonies are organized into various naturalistic social groups, typically comprising multiple matrilines, with adult females and their offspring, accompanied by a single non-natal adult male. From the population of Indian-origin rhesus macaques, 44 individuals were included in the study, comprising 37 animals from four families and seven unrelated males and females (**Supplementary Table 1**, **Supplementary Figure 1**). In addition, samples from 17 Burmese-origin rhesus macaques (three families and three unrelated males and females) and 17 Chinese-origin (three families and seven unrelated individuals) from the BPRC biobank were selected for inclusion (**Supplementary Table 1**, **Supplementary Figure 1**). The BPRCs long-tailed macaque colony represents a mixed population based on geographic origin, comprising animals from regions both north and south of the isthmus of Kra, and from the Indonesian and Malaysian islands (40, 42). From this colony, 70 animals were selected, comprising eight families and 9 unrelated males and females (**Supplementary Table 1**, **Supplementary Figure 1**).

Twenty-nine Hamadryas baboon samples, comprising 24 individuals from five families and five unrelated individuals, were selected from our biobank for inclusion in the study (**Supplementary Table 1**, **Supplementary Figure 1**). These samples originate from a cohort of Hamadryas baboons living at WILDLANDS Adventure Zoo in Emmen, The Netherlands.

Four anonymized, family-related human PBMC samples collected in the early 1980s, originating from a hematologic patient whose family had been typed, were kindly provided by the Immunohematology and Blood Transfusion department of the Leiden University Medical Centre. These samples were used to characterize *LILRB1* transcriptome polymorphism.

### Genomic DNA isolation, RNA extraction and cDNA synthesis

During regular health checks, heparin and/or ethylenediaminetetraacetic acid (EDTA) samples were obtained from the BPRC-housed rhesus and long-tailed macaques and served as a source for DNA and RNA. Genomic DNA (gDNA) was extracted from EDTA blood or isolated PBMCs using a standard salting-out procedure. RNA was extracted from EDTA blood using the RNeasy mini kit (Qiagen, Hilden, DE) and was used as input for the synthesis of cDNA by the RevertAid first strand cDNA synthesis kit (Invitrogen, Carlsbad (CA), USA) according to manufacturer’s instructions.

### *LILRB1* and *LILRB2* transcriptome amplification and SMRT sequencing on PacBio platform

To obtain *LILRB1* and *LILRB2* amplicons, gene-specific primers were designed (**Supplementary Table 2**). The primer set *Mamu-LILRB1*-Fw1-Rv1 was used for all rhesus macaque samples, and the products amplified with this set resulted in *LILRB1* transcripts of 2077 base pair (bp) in length, spanning from exon 4 to the 3’UTR. The primer set *Mamu-LILRB1*-Fw2-Rv2 was used for a subset of rhesus macaque samples to generate *LILRB1* transcripts of 2533 bp in length, spanning from exon 3 to the 3’UTR. For the long-tailed macaques, one forward (Fw) and one reverse (Rv) primer were designed, both located within the transcript (Fw in exon 3 and Rv in exon 16), resulting in the amplification of *LILRB1* from exon 4 to partially exon 16 (108 out of the 147 bp of exon 16) and a total product size of 1761 bp. This latter primer set was also used to amplify *LILRB1* for the Hamadryas baboon samples. To amplify *LILRB1* transcripts in the human samples, the primer set *Hosa-LILRB1*-Fw-Rv was used, generating a product of 2405 bp. A generic primer set was designed to amplify *LILRB2* transcripts for rhesus macaque, long-tailed macaque, and Hamadryas baboon samples, spanning 1788 bp from exon 4 to the 3’UTR. The PCR reactions (50 μl) contained 5 μl of cDNA, 1x Phusion HF buffer, 0.2 mM dNTPs, 0.5 μM of the forward and reverse primer, 3% DMSO, and 0.02 U/μl Phusion hot start II DNA polymerase (Thermo Fisher Scientific, Waltham (MA), USA). Each primer was tagged at the 5’end with a unique 16 bp barcode (www.pacb.com) to allow identification of the pooled amplicons from the different samples after SMRT sequencing on a PacBio platform. For amplification of *LILRB1* and *LILRB2*, thermal cycling conditions started with a denaturation step at 98°C for 2 min, followed by 35 cycles of 98°C for 10 s, 60°C or 62°C for 40 s (annealing temperature applied is specified in **Supplementary Table 2**), 72°C for 40 s, and a final extension at 72°C for 10 min. The amplification of human *LILRB1* started with a denaturation step at 98°C for 2 min, followed by 35 cycles of 98°C for 10 s, 60°C for 40 s, 72°C for 180 s, and a final extension at 72°C for 10 min. Gel electrophoresis was used to size select PCR products (**Supplementary Figure 2**), and amplicons were purified using GeneJet gel extraction kit (Invitrogen, Carlsbad (CA), USA) according to manufacturer’s instructions. DNA concentrations of individual amplicons were quantified using the Qubit dsDNA HS assay kit and Qubit 2.0 Fluorometer (Thermo Fisher Scientific, Waltham (MA), USA). Amplicons were then pooled in equimolar amounts to a total yield of 3 μg of DNA. The pooled samples were purified twice using AMPure XP beads (Beckman Coulter, Brea (CA), USA) at a 1:1 bead to DNA volume ratio according to manufacturer’s instructions. After purification, the DNA of the pooled samples was re-measured to ensure a total yield of >1 μg total DNA. The PacBio SMRTbell libraries were generated according to Pacific Biosciences “Procedure & Checklist – Amplicon Template Preparation and Sequencing” and sequencing was performed at the Leiden Genome Technology Centre using a PacBio Sequel I (P6-C4 sequencing chemistry) or Sequel II (sequencing kit versions 2.0 and 2.1) system (39). Sequence data collection was performed with a 10-hour (Sequel I) or 20-24-hour (Sequel II) movie time window to obtain sufficient yields of high-quality circular consensus reads. In total, ten runs were performed on a Sequel I and eleven on a Sequel II system to characterize and confirm the various *LILRB1* and *LILRB2* alleles at the transcription level in the human, two macaque, and Hamadryas baboon samples (**Supplementary Table 3**). The average number of reads per sample varied from 2736 to 29543. Two Sequel I runs contained only four samples, and the average read count was 28074 and 34581 (**Supplementary Table 3**).

### *LILRB1* and *LILRB2* transcriptome data analysis

Circular consensus sequences (CCS) were filtered for high read quality (value of 0.99 or higher) and demultiplexed based on unique barcodes. The “Map to reference” function (setting Geneious mapper) in the Geneious Prime version 2025.0.3 was used to align the CCS reads to either a *LILRB1* or *LILRB2* reference library. Reads with a 100% match were identified using the following settings: fine tuning iterate up to 5 times, minimum overlap = 400, maximum mismatches per read 0, 100% minimum overlap identity, maximum ambiguity = 1. Reference libraries were established per species. The *LILRB1* reference libraries consisted of previously reported transcripts for humans and rhesus macaques (12, 43), supplemented with directly submitted as well as predicted reference *LILRB1* sequences for humans, rhesus and long-tailed macaques (**Supplementary Table 4**). The construction of the *LILRB2* reference library followed a different strategy. Through comparative analyses of genomic *LILR* organizations (5), we identified LOC102143922, designated *Mafa-LILRA3* in the Gene database (https://www.ncbi.nlm.nih.gov/genbank/), as *Mafa-LILRB2*. In addition, among the reads amplified with the *LILRB1* primers, a group of deviating alleles was identified, which appeared to cluster phylogenetically with LOC102143922. These alleles, along with the sequence of LOC102143922, were used as the reference library to further characterize *LILRB2* alleles in macaque species. For baboons, comparative analyses showed that LOC101022334 (*Paan-LILRA3* in the Gene database) and LOC116271753 (*Paan-LILRB1* in the Gene database) are identified as *Paan-LILRB1* and *Paan-LILRB2*, respectively. Following the initial analyses, additional reference libraries were constructed for human, rhesus and long-tailed macaques, and Hamadryas baboons incorporating alternatively spliced *LILRB1* and *LILRB2* transcripts based on data generated in this study. Unused reads were grouped into contigs based on similarity using Geneious software, and the consensus sequence of each contig was compared with the *LILRB1* and *LILRB2* transcript reference library and analyzed through phylogenetic analysis. The novel sequences/alleles (cutoff value of three reads per contig) were confirmed by identification in two independent PCRs and PacBio runs and/or identified in at least two individuals.

### *LILRB1* and *LILRB2* gDNA amplification, ONT and PacBio SMRT sequencing, and data analysis

Representative rhesus and long-tailed macaque families (14 and 7 individuals, respectively, see **Supplementary Table 1**), which were also included in the transcriptome analysis, were selected for *LILRB1* characterization at the gDNA level using long-read sequencing on an ONT platform. The PCR reactions (50 μl) to amplify genomic *LILRB1* was performed with 250 ng gDNA (50 ng/μl) as input, 1x Phusion HF buffer, 0.2 mM dNTPs, 0.5 μM of each forward and reverse primer (**Supplementary Table 2**), 3% DMSO, and 0.02 U/μl Phusion hot start II DNA polymerase. This primer set (Fw-Rv) amplified a 5787 bp product that spans from intron 3 to part of exon 16 (107 out of the 147 bp of exon 16). The thermal cycling conditions consisted of a denaturation step at 98°C for 2 min, followed by 35 cycles of 98°C for 10 s, 62°C for 30 s, 72°C for 4 min, and a final extension at 72°C for 10 min. Gel electrophoresis and extraction were performed as described above. Individual amplicons were barcoded using the ONT Native Barcoding Expansion kit (EXP-NBD103, Oxford Nanopore Technologies Ltd, Oxford, UK), and pooled equimolar (12 samples per pool). ONT sequencing adapters were subsequently ligated to the pooled barcoded amplicons using the Ligation Sequencing kit (SQK-LSK109, Oxford Nanopore Technologies Ltd) according to the ONT Amplicons by Ligation protocol (version protocol NBA_9093_v109_revF_12Nov2019; ligation kit LQK-LSK109). Library clean-up was performed with AMPure XP beads and ONT Long Fragment Buffer to remove ligation enzymes and to enrich for >3 kb transcripts. A MinION R9.4.1 flow cell (Oxford Nanopore Technologies Ltd) was primed using the Flow Cell Priming kit (EXP-FLP002) prior to sequencing. Flow cells were run for up to 24 hours. The reads were demultiplexed and base called using Guppy V3.4.1 software and analyzed with Geneious.

In addition, gDNA sequences of the *LILRB1* and *LILRB2* alleles were characterized using SMRT sequencing on a PacBio platform. PCR reactions (50 μl) were performed using 250 ng of gDNA (50 ng/μl) as input, 1x Phusion HF buffer, 0.2 mM dNTPs, 0.5 μM of each forward and reverse primer (**Supplementary Table 2**), 3% DMSO, and 0.02 U/μl Phusion hot start II DNA polymerase. For *LILRB1*, two sets of primers were used, the first set amplified a 5787 bp product as described above and the second set (Fw3-Rv3) a 5739 bp product that spans intron 3 through part of exon 16 (45 out of the 147 bp of exon 16). For *LILRB2*, the primers amplified a 5887 bp product that spans from the start codon located at the end of exon 3 to the 3’UTR. The primer sets were barcoded as described for the cDNA amplification. The thermal cycling conditions consisted of a denaturation step at 98°C for 2 min, followed by 35 cycles of 98°C for 10 s, 60 or 62°C for 40 s (**Supplementary Table 2**), 72°C for 6 min, and a final extension at 72°C for 10 min. Gel electrophoresis and amplicon extraction, pooling and sequencing of the samples were performed similar as described for the cDNA samples. Twelve runs were conducted on the Sequel II system to characterize and confirm the *LILRB1* and *LILRB2* alleles at the gDNA level (**Supplementary Table 3**). Data analyses were performed as previously detailed, using reference libraries supplemented with gDNA sequences extracted from the reference genomes of rhesus and long tailed macaque and subsequently expanded with the newly characterized alleles. Novel sequences/alleles were confirmed by identification in two independent PCRs and PacBio runs and/or by their detection in at least two individuals. All genomic *LILRB1* and *LILRB2* sequences characterized in this study have been submitted to the ENA-EMBL database under project number PRJEB86133 and received individual accession numbers (**Supplementary Table 5**). In addition, seven alleles were identified only at the transcriptional level and were submitted under the same project number.

### Confirmation of 21bp insert exon 9 by Sanger sequencing

PCR (50 μl) amplification to confirm the presence of the 21 bp insert in specific alleles/lineages was performed on cDNA (5 μl) with the primer set LILR-ex7-Fw and LILR-ex7-Rv (**Supplementary Table 2**) and using Phusion hot start II DNA polymerase. The thermal cycling conditions consisted of a denaturation step at 98°C for 2 min, followed by 32 cycles of 98°C for 10 s, 62°C for 15 s, 72°C for 2 min, and a final extension at 72°C for 10 min. Gel electrophoresis and amplicon extraction were performed as described above. Samples were directly sequenced in house on an ABI 3130xl genetic analyzer (Applied Biosystems, Foster City, CA) using the ABI Prism BigDye Terminator v3.1 Cycle sequencing mixture (Applied Biosystems). The data were analyzed using MacVector version 18.7.6 (MacVector, Inc Cambridge, UK).

### Phylogenetic analysis

Phylogenetic analysis was performed in Geneious prime (version 2025.0.3) using the neighbor-joining method (44), and two models for computing the evolutionary distances: Jukes-Cantor (45) and Tamura-Nei (46). Both genetic distance models produced trees with similar topologies. Subsequently, a neighbor-joining tree was constructed with MEGA 11 application (47) and the evolutionary distances were computed using the Nei-Gojobori (Jukes-Cantor) method (48) and are in the units of the number of nonsynonymous substitutions per nonsynonymous site. Bootstrap values were calculated based on 1,000 replicates (49).

Notably, in rhesus macaques, LILRB1- and LILRB2-like molecules, designated as Mm-LILRBa and MmLILRBb, respectively, were identified based on the screening of cDNA libraries derived from different tissues (12). Phylogenetic comparisons of the sequences of Mm-LILRBa (DQ155431) and MmLILRBb (DQ155432) with the current dataset showed that both Mm-LILRBa and MmLILRBb cluster within the LILRB1 group, with the sequence of Mm-LILRBa being identical to Mamu-LILRB1*001:01, and MmLILRBb clustering with macaque LILRB1*002 lineage alleles.

### LILRB1 and LILRB2 allele nomenclature in OWM species

Currently, no official allele nomenclature exists for *LILRB1* and *LILRB2*. Therefore, we propose a *LILRB1* and *LILRB2* allele nomenclature system for OWM species, developed based on the framework established for MHC nomenclature in primate species (50–52). To illustrate, we use the designation of the rhesus macaque allele Mamu-LILRB1*001:01:01:01 as an example. The four-letter prefix (in this case *Mamu*) defines the taxonomic group and is derived from the species’ scientific name: the first two letters from the genus (*Macaca*) and the last two letters from the species (*mulatta*). This is followed by a hyphen, the gene name (*LILRB1* in this example), and an asterisk. After the asterisk, the first field consists of three digits and represents the lineage, defined as a group of alleles clustering together in phylogenetic analyses. The second, third, and fourth fields, each composed of two digits, denote the allelic variation, synonymous mutations within the coding region, and differences in the non-coding region, respectively. Colons are used to separate these fields. The suffix “Sp” is added when the allele described is a splice variant.

## Results

### Characterization of the *LILRB1* and *LILRB2* transcriptomes in rhesus macaques, long-tailed macaques, and Hamadryas baboons

**Figure 1:**
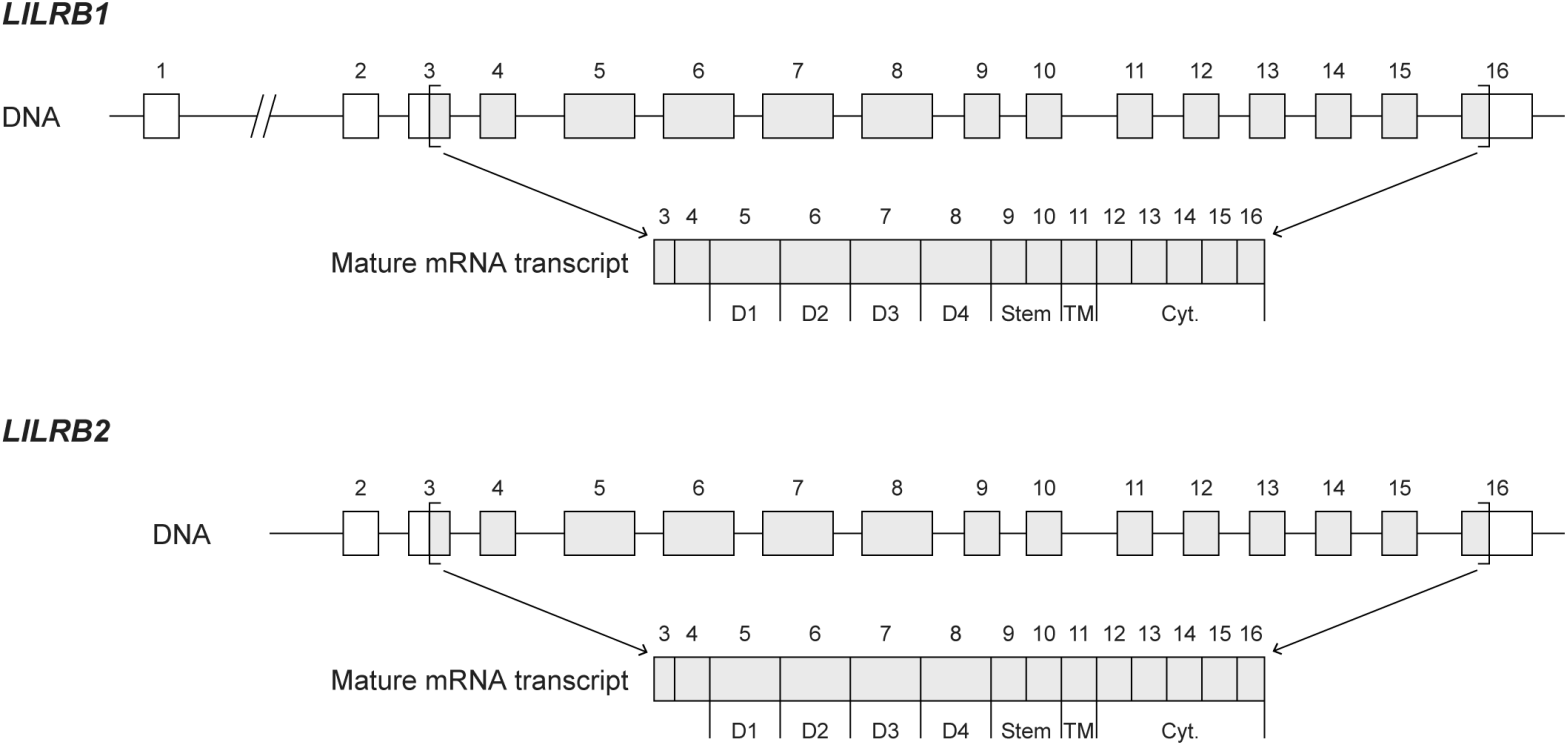
Schematic organization of the *LILRB1* and *LILRB2* gene. The upper panels illustrate the exon-intron structure of each gene, with exons represented as numbered blocks and introns as horizontal lines. In *LILRB1*, the break marked by two vertical lines represents an approximately 13 kb stretch of DNA (53). In the publicly available genome assemblies, exon 1 and the adjacent 13 kb intron 1 region present in *LILRB1* could not be clearly identified for *LILRB2*, and therefore only exon 2 through the 3’UTR region is shown. Exons representing the mature mRNA *LILRB1* and *LILRB2* coding sequences are indicated by gray-colored blocks. The structural features of the LILRB1 and LILRB2 molecules encoded by the various exons are labeled, with D1 to D4 representing the four extracellular domains, “TM” referring to the transmembrane region, and “Cyt.” referring to the cytoplasmic tail.

The *LILRB1* gene consists of 16 exons, and the corresponding mature mRNA is encoded by 14 of these exons. The start codon is located in exon 3, and the stop codon at the beginning of exon 16, after 147 bp (**Figure 1**) (53). For *LILRB2*, our sequencing results indicate a comparable exon-intron organization across the coding region of the gene. The four Ig-like domains (D1 to D4) of LILRB1 and LILRB2 are encoded by exons 5-8, while exons 9 and 10 encode the stem (spacer) region, exon 11 encodes the transmembrane segment, and exons 12–16 encode the cytoplasmatic tail.

To characterize the polymorphism of *LILRB1* and *LILRB2* genes in macaques, samples were selected from both related and unrelated individuals within the pedigreed macaque cohorts housed at the BPRC (**Supplementary Figure 1**). Additional rhesus macaque samples of Burmese and Chinese origin were included. In total, 74 rhesus macaques (44 Indian, 15 Burmese, 15 Chinese), along with 62 long-tailed macaques, and 29 Hamadryas baboon samples were examined. Transcriptome analysis of the *LILRB1* and *LILRB2* revealed that most reads represented alternatively spliced transcripts, while only a minority corresponded to the mature mRNA. Overall, the transcriptome analyses resulted in the identification of 93 *LILRB1* and *LILRB2* alleles across the three species (**Table 1**, **Supplementary Table 5**). Of these, only one allele, Mamu-LILRB1*001:01, had been previously reported (12). Notably, all four baboon *LILRB1* alleles, and several macaque *LILRB1* alleles predominantly lacked exon 15 in their transcripts, most likely due to alternative splicing, and are therefore designated with the abbreviation *Sp* (**Supplementary Table 5**, alleles marked with a $ sign). Similarly, some *LILRB2* alleles predominantly lacked exon 9 at the transcriptome level (**Supplementary Table 5**, alleles marked with a # sign). One baboon allele, Paha-LILRB2*013:01Sp, was identified as splice variant containing a three base pair insertion along with the deletion of exon 9. These findings highlight the challenges of fully characterizing *LILRB1* and *LILRB2* polymorphisms using transcriptomic data alone. To address this, selected animals from each cohort were further analyzed at the gDNA level.

**Table 1:** Overview of the number of *LILRB1* and *LILRB2* alleles characterized in the rhesus macaque, long-tailed macaque, and Hamadryas baboon cohorts.

Species	Gene	# alleles cDNA level	# alleles gDNA level
Rhesus macaque	*LILRB1*	21	32
	*LILRB2*	13	26
Long-tailed macaque	*LILRB1*	28	39
	*LILRB2*	20	29
Hamadryas baboon	*LILRB1*	4	5
	*LILRB2*	7	4

Detailed information on the alleles and the animals analyzed can be found in **Supplementary Tables 1** and **5**.

### Genomic characterization of *LILRB1* and *LILRB2* in macaques and Hamadryas baboons: phylogeny and lineage diversification

To characterize the *LILRB1* and *LILRB2* allelic polymorphisms present at the genomic level in our three cohorts of OWM, we selected 30 Indian, 13 Burmese, and 16 Chinese rhesus macaques, 54 long-tailed macaques, and 14 Hamadryas baboons (**Supplementary Table 1**). Most of these samples were sequenced using the PacBio platform, however, a small subset was sequenced using the ONT workflow. The analyses resulted in the identification of 135 *LILRB1* and *LILRB2* alleles across the three species (**Table 1**, **Supplementary Table 5**). This total included 86 alleles previously identified at the transcription level, now extended with intronic sequence information, and 49 newly detected alleles.

**Figure 2:**
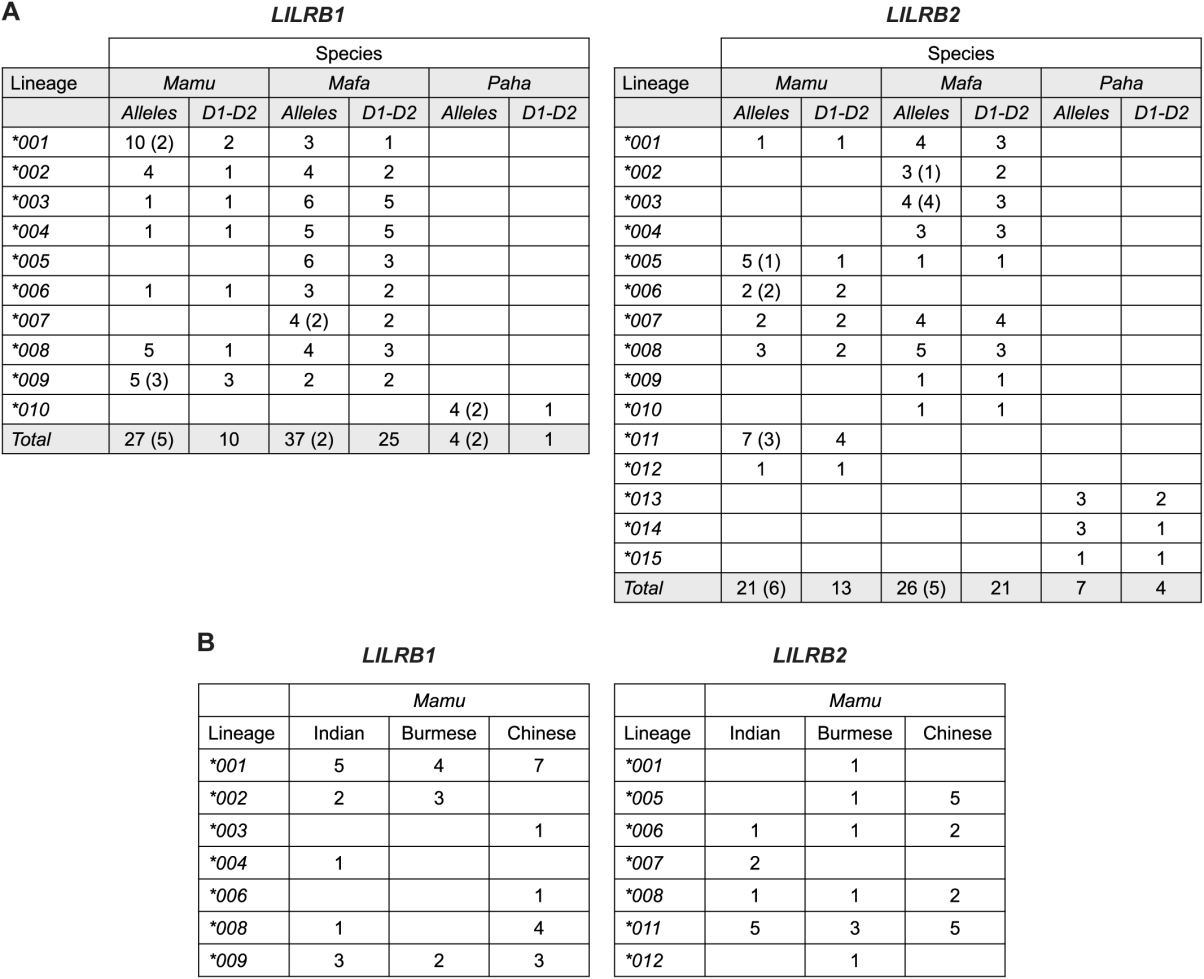
Overview of the *LILRB1* and *LILRB2* lineages in rhesus (*Mamu*) and long-tailed (*Mafa*) macaques and Hamadryas baboon (*Paha*). **(A)** For each species, the number of alleles identified per lineage is shown, with the number of alleles exhibiting intron-level variation indicated in parentheses. The number of distinct D1-D2 amino acid sequences translated from these alleles is also provided (additional supporting data is provided in **Supplementary Table 7**). **(B)** Lineage distribution across the rhesus macaque populations, categorized by geographic origin (Indian, Burmese, and Chinese), with the number of alleles per lineage indicated.

Subsequently, we performed phylogenetic analysis to explore the evolutionary relationships among *LILRB1* and *LILRB2* alleles in the three species and in humans (**Supplementary Figure 3**). This analysis showed that human *LILRB1* and *LILRB2* sequences cluster separately from those of OWM. Alleles from the two macaque species intermingled across several distinct clusters, while the Hamadryas baboon sequences formed species-specific branches. Based on sequence alignments and phylogenetic clustering patterns, we defined 10 *LILRB1* and 15 *LILRB2* lineages to describe the sequence variation observed across the three OWM species (**Figure 2A**, **Supplementary Figure 3**). For naming the OWM *LILRB1* and *LILRB2* alleles (**Supplementary Table 5**), we applied the nomenclature proposed in the *Materials & Methods* section.

All *LILRB1* lineages are supported by four or more alleles. For *LILRB2*, 11 lineages are supported by three to ten alleles, while four lineages currently contain a single allele (**Figure 2A**). Most *LILRB1* lineages are shared between the two macaque species, except for *005 and *007, which were unique to long-tailed macaques. In contrast, less sharing is observed for *LILRB2* lineages (only 4 out of 12), with three and five distinct lineages specific to rhesus and long-tailed macaques, respectively (**Figure 2A**). In Hamadryas baboons, lineage diversity was limited, with alleles clustering into one *LILRB1* and three *LILRB2* lineages (**Figure 2A**, **Supplementary Figure 3**).

For the rhesus macaque, we distinguished animals of Indian, Burmese, and Chinese origin. Several lineages were shared across these geographical populations (**Figure 2B**). Furthermore, sharing between Indian and Burmese, Indian and Chinese, and Burmese and Chinese animals were observed for LILRB1*002, LILRB1*008, and LILRB2*005, respectively (**Figure 2B**). The remaining lineages were restricted to a single population. A few alleles were pairwise shared between the different rhesus macaque populations, whereas only Mamu-LILRB1*001:04:01:01 was common to all three rhesus macaque populations (**Supplementary Table 5**). These results indicate that most rhesus macaque *LILRB1* and *LILRB2* alleles are population-specific, likely reflecting historical geographic barriers and limited gene flow. Furthermore, no allelic sharing of *LILRB1* and *LILRB2* was observed between rhesus and long-tailed macaques, suggesting further diversification following speciation.

### Sequence characteristics and polymorphic sites of *LILRB1* and *LILRB2*

Alignments of the *LILRB1* and *LILRB2* coding sequences from rhesus macaques, long-tailed macaques, and Hamadryas baboons revealed both nucleotide polymorphisms and structural variations (**Supplementary Figure 4**). For example, insertions were identified in both *LILRB1* and *LILRB2*. In *LILRB1*, a 21 bp insertion mapping to exon 9 was observed in all currently described Mafa-LILRB1*005 alleles, as well as in Mafa-LILRB1*004:01:01:01, *007:03:01:01, and *007:04:01:01. This insertion results in an extension of the stem region by seven amino acids, and its presence was confirmed by Sanger sequencing. In *LILRB2*, a three bp insertion at positions 82–84 characterizes the macaque lineages *001, *002, *003, and *010. Another structural variation we observed involves the absence of exon 9 in certain *LILRB2* alleles, resulting in a shortened stem region. This likely represents alternative spliced variants, as in case of Mafa-LILRB2*002:02 and *008:04 exon 9 could be identified at the gDNA level (**Supplementary Table 5**).

**Figure 3:**
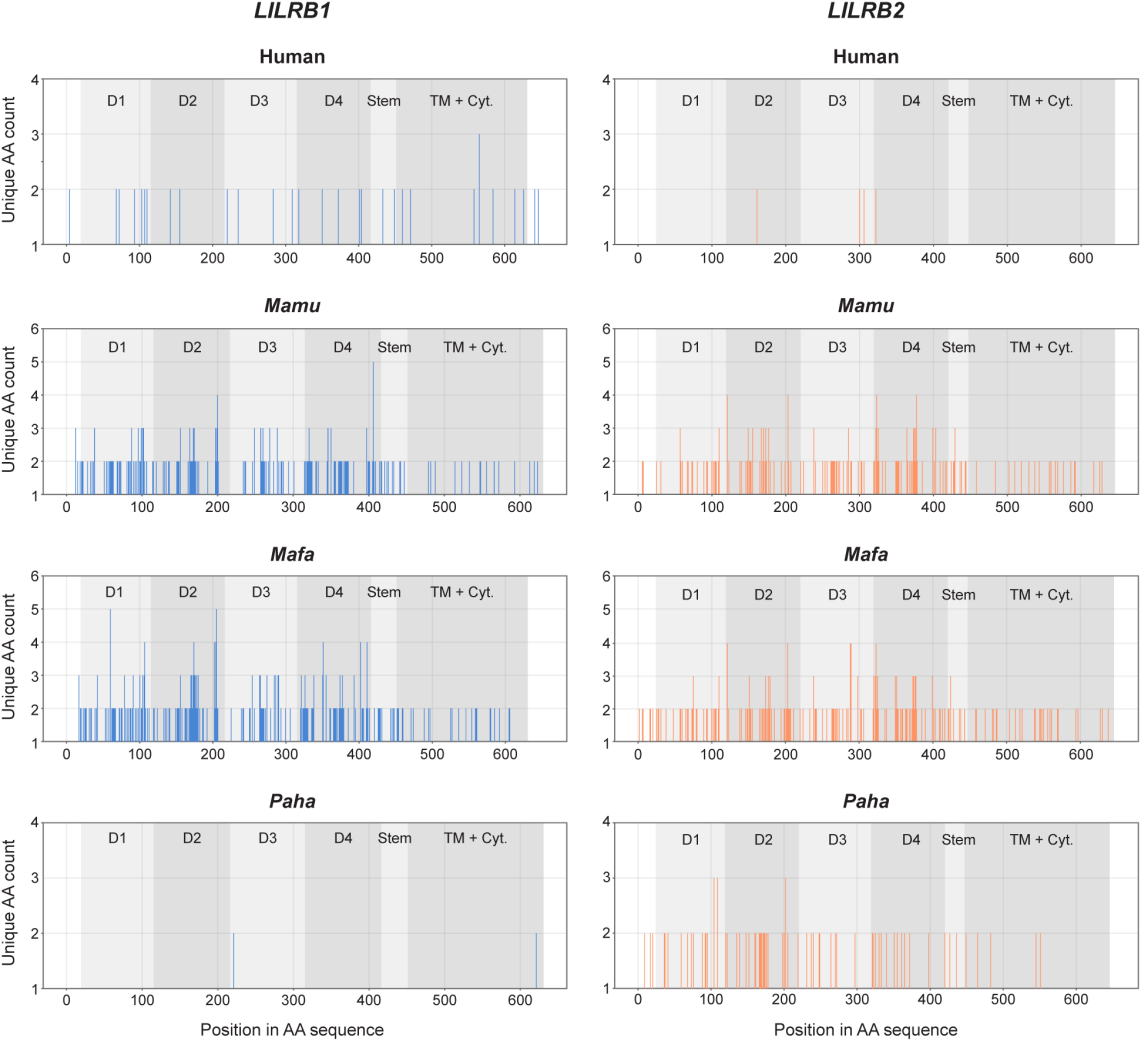
Variability plots of *LILRB1* and *LILRB2* of human, rhesus (*Mamu*) and long-tailed (*Mafa*) macaques, and Hamadryas baboon (*Paha*). The x-axis sequentially numbers the amino acids positions in the sequence alignment. The y-axis indicates the total number of unique amino acids (AA) encountered at a certain position. Source information for the human sequences used for comparison is provided in **Supplementary Table 4**.

**Figure 4:**
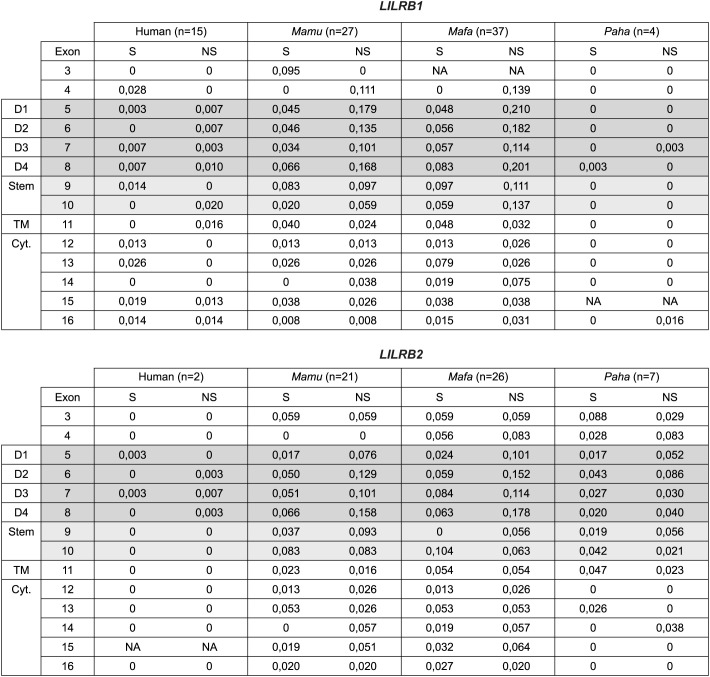
Overview of the synonymous (S) and non-synonymous (NS) mutations in *LILRB1* and *LILRB2* alleles from rhesus (*Mamu*) and long-tailed macaques (*Mafa*), and Hamadryas baboons (*Paha*). Mutation counts are normalized to exon length, with exon lengths and raw S/NS counts provided in **Supplementary Table 6**. For comparison, data from human *LILRB1* and *LILRB2* alleles are included. These human alleles correspond to those listed in **Supplementary Table 4**. “NA” indicates not applicable and is used when the corresponding part of the sequence was not characterized or when the exon was not detected/characterized. Distinct grey backgrounds highlight the data corresponding to the four domain (D) regions and the stem region. “TM” refers to the transmembrane region, and “Cyt.” to cytoplasmic tail region.

Next, the deduced amino acid sequences of the reported human *LILRB1* alleles (**Supplementary Table 4**) were compared with those of the rhesus macaque, long-tailed macaque, and Hamadryas baboon *LILRB1* alleles characterized in this study. Variability plots showed that non-synonymous polymorphisms, resulting in amino acid changes, are distributed throughout the coding sequence in all four species (**Figure 3**). In the Hamadryas baboon population, four *LILRB1* alleles were identified with a high degree of sequence similarity, exhibiting only two amino acid-changing sites, one in the D3 and one in the cytoplasmic tail (**Figure 3**). In contrast, rhesus and long-tailed macaques exhibit more diversity, with 27 and 37 *LILRB1* alleles, respectively, and an extensive number of non-synonymous changes, particularly within the exons that encode the Ig-like domains (D1-D4). Normalization to exon length confirmed elevated non-synonymous mutation density in these domains in macaques as compared to humans and Hamadryas baboon (**Figure 4**, **Supplementary Table 6**). Exons 4 and 10 of macaque *LILRB1* also exhibited increased non-synonymous variation.

For *LILRB2*, macaques displayed a similar pattern to that observed for *LILRB1*, with polymorphisms concentrated in the domain-encoding exons (**Figures 3**, **4**). In Hamadryas baboons, greater *LILRB2* diversity relative to *LILRB1* was observed. Notably, only two human *LILRB2* sequences are currently available (**Supplementary Table 4**), limiting comparisons across species.

### Sequence sharing in the D1 and D2 domains of *LILRB1* or *LILRB2* between rhesus and long-tailed macaques

Mature mRNA *LILRB1* or *LILRB2* coding sequences are not shared between rhesus and long-tailed macaques. However, within lineages, macaque *LILRB1* and *LILRB2* alleles can exhibit high sequence similarity. To assess the potential impact of the polymorphisms encountered in *LILRB1* and *LILRB2* on MHC class I recognition, we compared the deduced amino acid sequences of exons 5 (D1) and 6 (D2) in the two macaque species and Hamadryas baboons.

**Figure 5:**
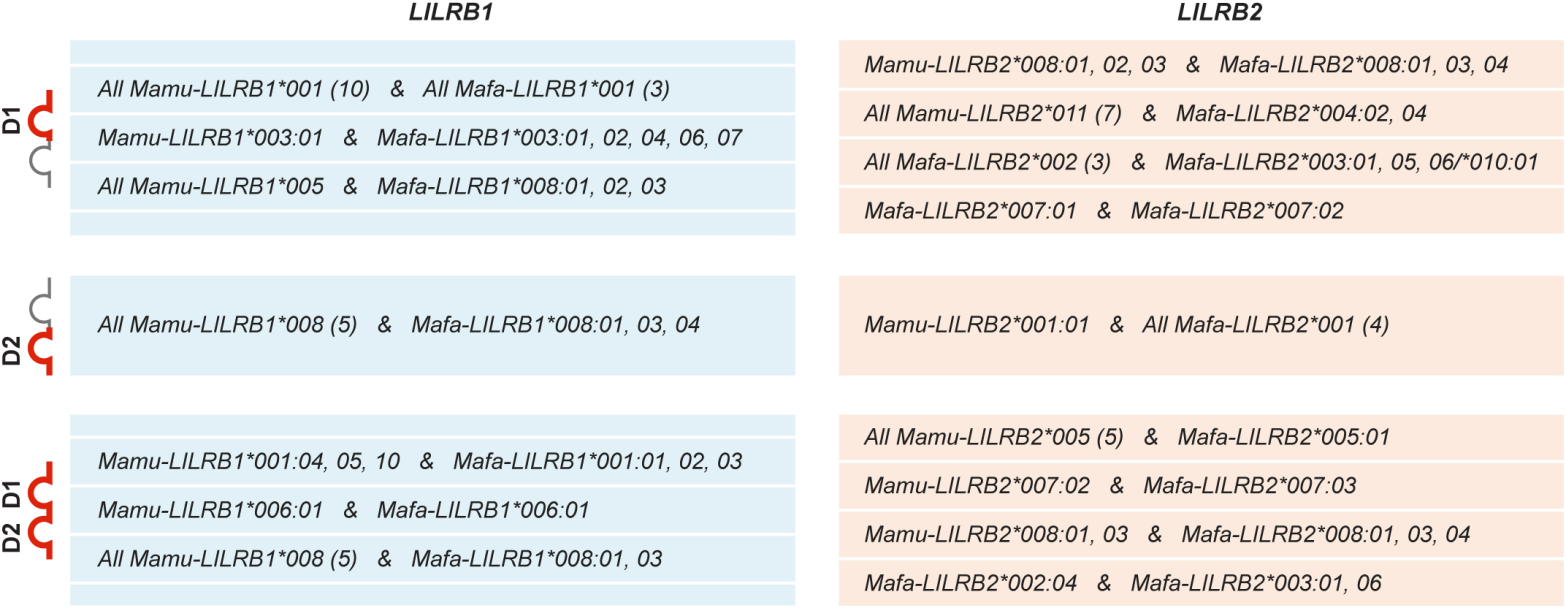
Rhesus (*Mamu*) and long-tailed (*Mafa*) macaque *LILRB1* and *LILRB2* alleles that share D1, D2, or combined D1-D2 amino acid sequences. Schematic illustrations on the left side of the figure represent the D1, D2, and combined D1-D2 domains, with adjacent each colored block (blue for *LILRB1*, light orange for *LILRB2*) indicating the combination of alleles sharing the same sequence for exon 5 (D1), exon 6 (D2), or exons 5 and 6 (D1-D2). For lineages in which all currently known alleles share the same corresponding domain sequence, the total number of alleles is shown in parentheses. When only a subset of alleles within a lineage shares the indicated domain sequence, the identifying digits of those alleles are listed next to the first allele name, separated by commas.

Alleles within a specific lineage may share the same D1-D2 amino acid sequence, and this was observed for all three species (**Figure 2A**, **Supplementary Table 7**). The greatest D1-D2 sequence variation was observed in long-tailed macaques, where the characterized nucleotide sequence diversity translated into 25 distinct D1-D2 amino acid sequences for *LILRB1* and 21 for *LILRB2* (**Figure 2A**). Across species, sharing of D1, D2, and combined D1-D2 amino acid sequence was observed among specific rhesus and long-tailed macaque *LILRB1* and *LILRB2* lineage alleles (**Figure 5**). Notably, D1 sequence sharing was detected among alleles of the Mamu-LILRB2*011 lineage and the Mafa-LILRB2*004:02 and *004:04 alleles. Although these alleles cluster closely in phylogenetic analyses (**Supplementary Figure 3**), we classified them into distinct lineages based on sequence differences in the D3, D4, stem, and cytoplasmic tail regions. Furthermore, sequence sharing of the D1 domain and the combined D1-D2 domains was identified in certain *LILRB2* lineages currently detected only in long-tailed macaques (**Figure 5**). Overall, these findings indicate that although an increased number of non-synonymous mutations is present in the *LILRB1* and *LILRB2* extracellular domain encoding regions (**Figure 4**), the D1 and D2 encoding regions −involved in MHC class I interaction− remain highly conserved within a lineage, and conservation for these domain encoding regions is even observed among lineages shared between rhesus and long-tailed macaque.

### Polymorphism of the MHC class I alpha-3 domain and its association with *LILRB1* and *LILRB2* D1 variation

**Figure 6:**
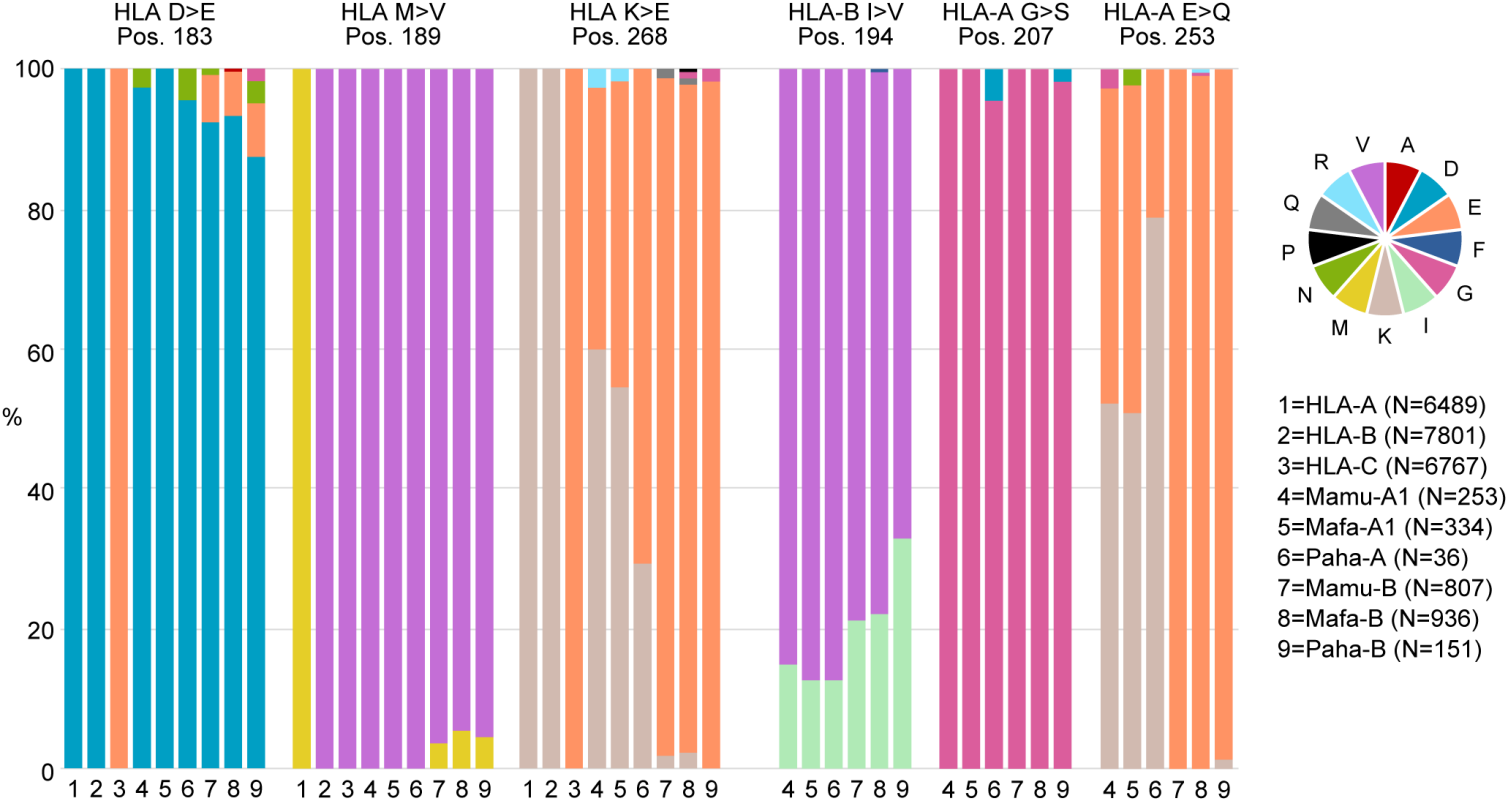
Stacked bar plots illustrating the distribution of amino acids at MHC alpha 3 domain positions associated with significant binding differences to LILRB1 in humans. Above each stacked bar plot the amino acid position (Pos.), the HLA type involved, and the amino acids associated with increased binding affinity in the HLA-LILRB1 study are shown (53). For human (HLA), rhesus macaque (Mamu), long-tailed macaque (Mafa), and Hamadryas baboon (Paha) the number (N) of complete exon 4 sequences analyzed is indicated. Distinct amino acids are represented using the conventional one-letter symbols. Additional supporting data are provided in **Supplementary Table 8**.

The crystal structure of the HLA-LILRB1 complex shows strong binding between β2M and the LILRB1 D2 domain, in concert with a more flexible interaction between the HLA-α3 domain and LILRB1 D1 (17, 18, 54). LILRB2, in contrast, has been shown to bind HLA-G in a β2M-independent manner (54). *In-vitro* studies demonstrated that LILRB1 binds HLA-C with significantly lower affinity compared to HLA-A and -B, and that polymorphisms within the α3 domain can modulate this interaction, likely through electrostatic effects (53). To assess the extent of the α3 domain diversity among human, macaque, and baboon *Mhc* class I alleles and its potential impact on the interaction with the D1 domains of LILRB1 and LILRB2, we compared the deduced amino acid sequences of exon 4 from *HLA-A*, *-B*, *-C* and *-G* alleles (IPD-IMGT/HLA Database release 3.60), as well as from *Mamu-A1* and *-B*, *Mafa-A1* and *-B*, and *Paha-A* and *-B* alleles (IPD-MHC NHP Database release 3.14.0.0) (**Supplementary Table 8A**). Notably, unlike humans, macaques and baboons lack an *HLA-C* equivalent and possess expanded and variable *Mhc-A* and *-B* gene repertoires per haplotype (50, 55–59). The comparison revealed relatively greater protein variation in the macaque and baboon MHC-A1 and -B α3 domains compared to the HLA-A, -B, -C, and -G α3 domains (**Supplementary Table 8A**). In HLA, six amino acid positions were associated with significant differences in binding affinity for LILRB1 D1-D2 variants (53) (**Figure 6**). At these positions, in macaque and baboon MHC-A and -B allotypes the predominant residues were 183D, 189V, 268E −although a higher frequency of 268K was observed in Mamu- and Mafa-A1−, 194V, and 207G. At position 253, most macaque and baboon MHC-B allotypes contained E, whereas MHC-A allotypes displayed either E or K (**Figure 6**). Furthermore, we identified six α3 domain residues −187T, 198E, 202R, 203C, 208F, and 210P− that are conserved across macaque and baboon MHC-A and -B allotypes (**Supplementary Table 8B**) and are located near the positions highlighted in **Figure 6**.

From the perspective of LILRs, studies in humans have shown that positively charged residues at positions 72 (R72Q) and 103 (H103D) in the LILRB1 D1 domain enhance binding to HLA class I molecules (53). In macaque and baboons, the deduced amino acid sequences of LILRB1 and LILRB2 revealed that these positions are also predominantly occupied by positively charged residues. An exception is position 72 in LILRB2 D1, which is not positively charged but instead contains the neutral amino acids serine (S) and leucine (L) (**Supplementary Table 7**).

### Extensive alternative splicing characterizes the *LILRB1* and *LILRB2* transcriptomes of rhesus and long-tailed macaques, and Hamadryas baboons

Alternative splicing is a form of post-transcriptional modification that occurs in approximately 95% of the human multi-exon genes (60, 61). The introduction of next-generation sequencing techniques has enabled and enhanced large-scale characterization of alternatively spliced transcripts, as demonstrated, for example, in the *KIR* gene cluster (38, 41, 62).

**Figure 7:**
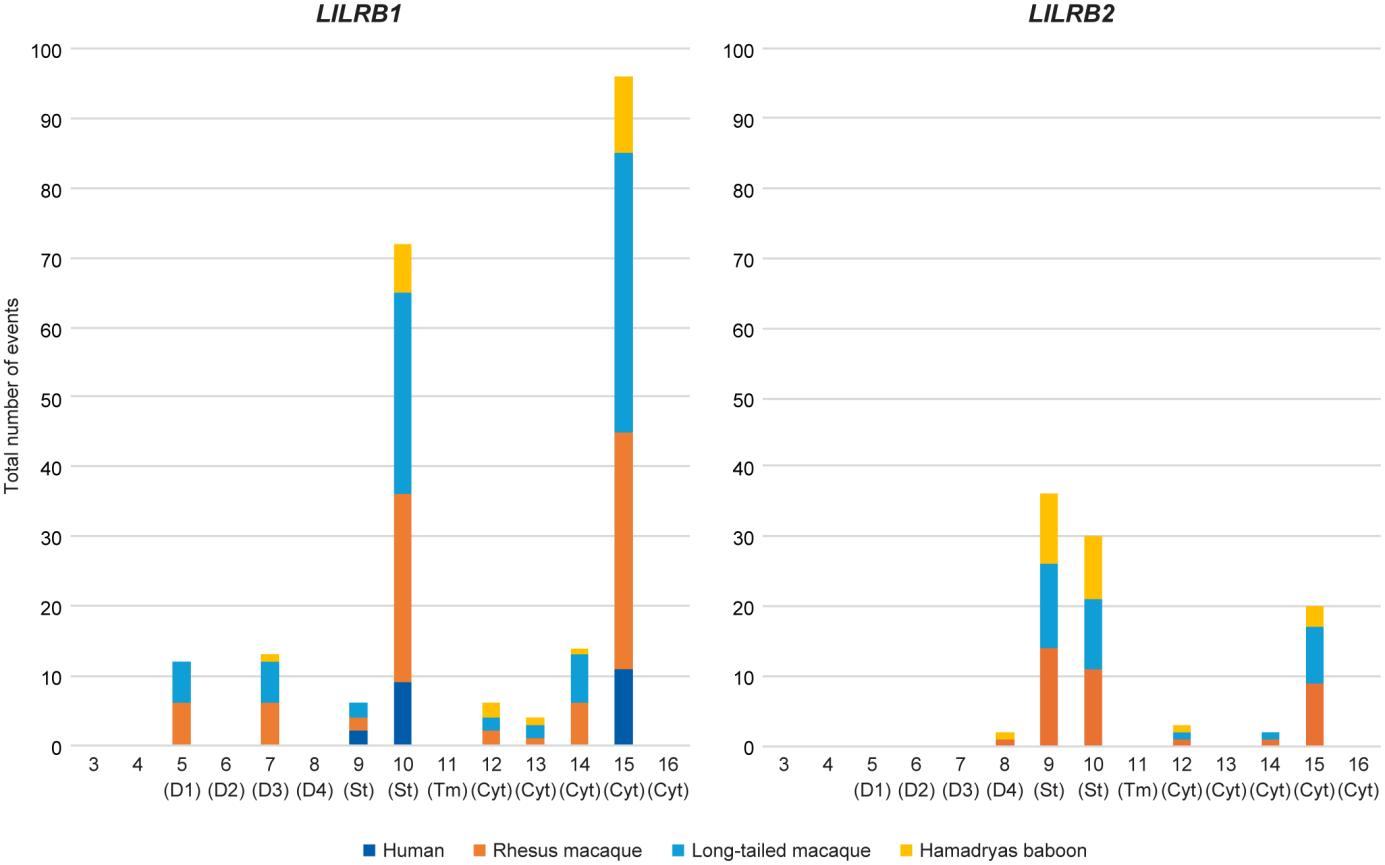
Stacked bar charts showing the absolute number of alternative splicing events involving the corresponding exon for *LILRB1* and *LILRB2* in humans, rhesus and long-tailed macaques, and Hamadryas baboons. Only alternative splicing events shared by at least two individuals/animals and present in two or more species were considered (**Supplementary Table 10**). On the x-axis, exons are numbered sequentially, as shown in **Figure 1**. D1-D4 indicate the four extracellular domains, “TM” denotes the transmembrane section, and “Cyt.” refers to the cytoplasmic tail.

**Figure 8:**
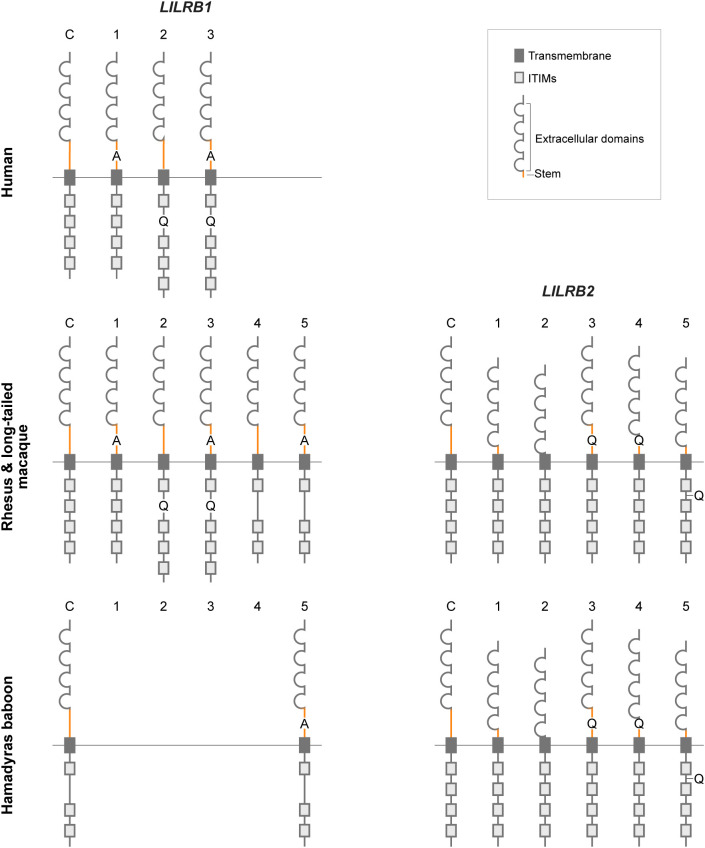
Schematic representation of the rhesus macaque, long-tailed macaque, and Hamadryas baboon LILRB1 and LILRB2 canonical (C) and alternatively spliced isoforms (numbered 1-5), as deduced from transcript sequences. For both LILRB1 and LILRB2, the deduced isoforms shown are the mature mRNA (C) or the result of the different splicing events discussed in the text (see **Supplementary Table 10**; events indicated in bold). These events may occur individually or in combination with other alternative splicing events (**Supplementary Table 10**). For human *LILRB2*, no transcriptome data were available. LILRB1: 1. 3 bp insertion in exon 10 (3’ss); 2. 3 bp insertion in exon 15 (3’ss); 3. 3 bp insertion in exons 10 and 15 (3’ss); 4. deletion of exon 15 (exon skipping); 5. 3bp insertion in exon 10 (3’ss) and deletion of exon 15 (exon skipping). LILRB2: 1. deletion of exon 9 (exon skipping); 2. deletion of exons 9 and 10 (exon skipping); 3. 3bp insertion in exon 10 (3’ss); 4. deletion of exon 9 (exon skipping) and 3bp insertion in exon 10 (3’ss); 5. deletion of exon 9 (exon skipping) and 3bp insertion in exon 15 (3’ss).

The transcriptome analysis of *LILRB1* and *LILRB2* in rhesus and long-tailed macaques and Hamadryas baboons revealed that transcripts were alternatively spliced (**Supplementary Tables 9A-D**). In *LILRB1*, the majority of the splice variants arose from a complex combination of alternative splicing events, predominantly involving exon skipping and the use of alternative 3’-splice site (ss) (**Supplementary Table 10A**). To compare splicing patterns across species, we also characterized the *LILRB1* transcriptome in four human samples (**Supplementary Figure 1**). In total, we identified 48 splicing events shared by at least two individuals/animals and present in two or more species (**Supplementary Table 10A**). Across all four species, alternative splicing affected exons 10 and 15, encoding the stem region and part of the cytoplasmic tail, respectively. These events were observed in the majority of splice variants (**Figure 7**) and involved different alleles (**Supplementary Table 10A**). One conserved event concerns an alternative 3’-ss in exon 10, resulting in a 3 bp insertion, leading to the addition of an alanine (A) residue in the stem region (**Figure 8**). This splicing event occurred in most, if not all, *LILRB1* alleles across all four species and is observed as independent event or in combination with other splicing events (**Supplementary Table 10A**). For exon 15, three common events were recorded. One involved an alternative 3’-ss near exon 15, resulting in a 3 bp insertion encoding a glutamine (Q) residue in the cytoplasmic tail region, an event shared by humans and macaques (**Figure 8**). The other two events included partial intron 15 retentions (typically around 30 bp, producing an exon 15 of 156 bp in length) and complete exon 15 skipping, the latter resulting in the loss of one of the four ITIM motifs present in the cytoplasmic tail of LILRB1 molecules (**Figure 8**). These events were observed in macaques and in both macaques and baboons, respectively. Exon 15 skipping is most likely explained by a GT-to-GC splice site mutation detected in all four Hamadryas baboon and most macaque *LILRB1* alleles (**Supplementary Table 5**). However, the presence of this splice site mutation does not invariably result in exon 15 exclusion. Also, some alleles contain an alternative splice site that may produce transcripts with an exon 15 of 126 bp in length. Additionally, small insertions of one or two base pairs at the start of exon 10, introducing premature stop codons, were detected across all four species for many different alleles (**Supplementary Table 10A**).

Next, the alternatively spliced *LILRB2* transcripts in rhesus and long-tailed macaques and Hamadryas baboons were investigated. Twenty splicing events were identified in at least two animals and across two species (**Supplementary Table 10B**). In many of these events (**Figure 7**) and in nearly all alleles (**Supplementary Table 10B**), exons 9, 10, and 15 were affected by alternative splicing. Deletion of exon 9, which may result in a shortened stem of LILRB2 (**Figure 8**), was commonly observed in all three non-human primate species, either as a single event or in combination with other splicing events (**Supplementary Table 10B**). One such combined event involved the deletion of exon 10, that may lead to the complete loss of the stem region in the LILRB2 structure (**Figure 8**). Other events involved the use of alternative 3’-ss near exons 10 and 15, resulting in the insertion of 3 bp encoding glutamine (**Figure 8**). These events were also detected either alone or in combination with other splicing events in numerous alleles across the three species (**Supplementary Table 10B**).

## Discussion

The immunomodulatory roles of LILRB1 and LILRB2 receptors make them promising targets for therapies in various types of cancer (63–66). In addition, infectious diseases such as tuberculosis and HIV, autoimmune diseases including multiple sclerosis and rheumatoid arthritis, and neurodegenerative conditions like Alzheimer’s disease may benefit from development of monoclonal antibodies that block the immune effector functions of these receptors (65). Although LILRB1 and LILRB2 have been studied in humans and, indirectly, in mice (which lack direct homologues of LILR), a detailed characterization in non-human primate species commonly used in preclinical research has been lacking. Here, we provide an in-depth genomic and transcriptomic analysis of *LILRB1* and *LILRB2* in rhesus and long-tailed macaques and Hamadryas baboons. Our results show that these genes are highly polymorphic and diversified in the macaque species but, similar to humans, are more conserved in Hamadryas baboons (**Figure 2**). Most polymorphisms are in the exons encoding the four extracellular domains (**Figures 3**, **4**). A detailed investigation of the deduced D1 and D2 amino acid sequences showed sharing of D1, D2, and combined D1-D2 sequences between the two macaque species (**Figure 5**), suggesting conserved ligand binding potential. Furthermore, we observed alternative splicing in *LILRB1* and *LILRB2* transcripts, involving most of the characterized alleles, with the majority of the splicing events affecting the exons encoding the stem and intracellular parts of the molecules (**Figure 7**). Several alternative splicing events are conserved across species, suggesting a possible role of these isoforms in regulating immunomodulatory responses.

The rather conserved nature of *LILRB1* and *LILRB2* in Hamadryas baboons, but also in humans, may indicate functional specialization. In contrast, the diversification of *LILRB1* and *LILRB2* in macaque species may reflect co-evolution with their highly diverse and expanded MHC class I system (67). Sequence comparison of the D1 and D2 domains, which are involved in binding the MHC class I α3 domain and β2M, between rhesus and long-tailed macaques revealed several identical and highly similar structures, suggesting conserved ligand binding in these species (**Figure 5**). However, the D1 and D2 domains may also contain species- and lineage-specific polymorphisms that are likely important for ligand interactions and the functional diversity of *LILRB1* and *LILRB2* in macaques. Analysis of the deduced amino acid sequences of the MHC-I α3 domain and the D1 domains of LILRB1 and LILRB2 showed that key residues involved in human LILRB1-MHC binding are also present in the three studied OWM species, suggesting potential for comparable interactions (**Figure 6**). At position 253 of macaque and baboon MHC-A allotypes, however, we observed oppositely charged residues, E (negatively charged) and K (positively charged), whose potential impact on LILRB1 and LILRB2 binding remains to be determined. Furthermore, within the α3 domain of the macaque and baboon MHC-A and -B allotypes, six conserved residues were identified located near key interaction sites (**Supplementary Table 8B**). Their conserved nature may indicate functional importance, for instance, in supporting stable binding to LILRB1 and LILRB2 in these species. Nonetheless, additional diversification at other positions within the macaque MHC-I α3 domain (**Supplementary Table 8B**) and within LILRB1 and LILRB2 (**Supplementary Table 7**) may influence binding characteristics and signaling potential, which effects need to be addressed in future functional studies.

Rhesus and long-tailed macaques are native to the Asian continent, whereas baboons are native to sub-Saharan Africa, where six distinct species are recognized (68). Among these, the Hamadryas baboon represents one of the smallest baboon populations and inhabits the Horn of Africa and the southwestern Arabian Peninsula. The condensed *LILRB1* and *LILRB2* repertoire that we identified in the Hamadryas baboons may be influenced by their limited population size. Therefore, additional research on *LILRB1* and *LILRB2* polymorphism in other baboon species is needed to determine whether the conserved nature of these genes is a general phenomenon in baboons. Alternatively, the differences in *LILRB1* and *LILRB2* diversity identified between the two macaque species and Hamadryas baboon may have been driven by their distinct habitats and the type of pathogens they encounter. For instance, macaques may have evolved *LILRB1* and *LILRB2* alleles that are specialized in recognizing decoy MHC class I molecules that are encoded by viruses, similarly to UL18 encoded by human cytomegalovirus (14, 19).

Within the *LILRB1* and *LILRB2* allelic repertoire, we identified alleles/lineages with in-frame insertions and deletions, mostly 3 bp events (**Supplementary Figure 4**). However, in case of the Mafa-LILRB1*005 lineage alleles, along with Mafa-LILRB1*004:01:01:01, *007:03:01:01, and *007:04:01:01, a 21 bp insertion in exon 9 was observed, extending the stem region with seven amino acids (PTTGPTS). The biochemical properties of these residues may influence the folding and stability of the LILRB1 molecule. For instance, threonine (T) and serine (S) can introduce hydrogen bonds, potentially stabilizing the LILRB1 structure, while glycine (G) −the smallest amino acid− may enhance flexibility, and proline (P) might introduce rigidity (69). The 21 bp insertion occurred in ~20% of the analyzed long-tailed macaque animals, including three homozygotes (**Supplementary Table 1**). Whether this insertion impairs receptor folding or instead enhances receptor function is yet undetermined.

Previously, our team reported on alternative splicing in the human and macaque *KIR* transcriptome (38). In humans, approximately 53% of *KIR2DL4* and 4% of *KIR2D/3D* transcripts were alternatively spliced, whereas in rhesus macaques, the corresponding proportions were approximately 13% and 24%, respectively. The *LILRB1* and *LILRB2* transcriptomes in rhesus and long-tailed macaques, and Hamadryas baboons exceed these levels, with the majority of reads representing alternatively spliced transcripts. For both *LILRB1* and *LILRB2*, alternative splicing primarily affected the stem region and part of the cytoplasmic tail involved in signal transduction (**Figure 7**). Skipping of exon 15 was observed in transcripts of *Mamu-*, *Mafa-*, and all *Paha-LILRB1* alleles. This event reduces the number of ITIM motifs from four to three and is likely associated with a GT-to-GC splice-site mutation. Although functional studies are required to assess the binding capacity and signal potential of isoforms, the abundance and conservation of specific alternative splicing events across species suggest that some may play an important role in modulating immune responses.

In humans, alternatively spliced soluble LILR isoforms have been described that are generated by the transcription of a cryptic stop codon located three nucleotides downstream of the donor splice site in the intron following the stem region (70). Human soluble LILRB1 has been shown to function as a negative regulator of the membrane bound counterpart, and a similar function is expected for other soluble LILRs. In macaque and Hamadryas baboon *LILRB1* and *LILRB2* alleles, a cryptic stop codon in the intron following the stem region was detected only in Mafa-LILRB1*009:02:01:01, Mafa-LILRB2*004:01:01:01, and the *Mamu-* and Mafa-LILRB2*008 lineage alleles. This suggests that the mechanism generating soluble isoforms through transcription of a cryptic stop codon, as seen in human *LILR*, may also occur in macaque *LILR*. However, most macaque and Hamadryas baboon *LILRB1* and *LILRB2* alleles lack a cryptic stop codon in this region. Instead, various 3’ splice site insertions of one to two base pairs immediately near exon 10 are found in macaque and baboon *LILRB1* and *LILRB2* transcripts. These insertions introduce premature stop codons and might result in the production of soluble isoforms.

In the HLA-LILRB1 complex it is demonstrated that binding between β2M and the D2 domain is crucial (17, 54). In case of the HLA-LILRB2 complex, β2M-independent binding has been observed (54), highlighting the importance of the interaction between the HLA class I α3 domain and the D1 domain of LILRB2. In none of the four species studied alternative splicing events were identified that affect the LILRB1 D2 domain (**Figure 7**). In case of LILRB2, the D1, D2, and D3 domains were exclusively constitutively spliced (**Figure 7**). These observations highlight the essential structural and functional roles of these domains in humans, both macaque species, and Hamadryas baboons.

This is the first comprehensive characterization of the *LILRB*1 and *LILRB2* repertoires in rhesus and long-tailed macaques and Hamadryas baboons. Previously we demonstrated that the primate *LILR* region has a highly conserved organization (5). Here we show that regarding *LILRB1* and *LILRB2* gene polymorphism and diversity, species specific differences exist. Given the role of macaque and baboon species as models for human diseases (27–29, 71), our findings provide a valuable foundation for advancing the interpretation and translation of immune modulatory responses. Future functional studies on *LILRB1* and *LILRB2* will be essential to fully address the implications of the polymorphisms and alternatively spliced variants identified in macaques and baboons.

## Data Availability

The datasets presented in this study can be found in online repositories. The names of the repository/repositories and accession number(s) can be found below: https://www.ebi.ac.uk/ena, OZ237016 till OZ237135, OZ237575 till OZ237580, OZ243064 till OZ243077, OZ243094, OZ243095.

## Supplementary material

Supplementary Figure 1 Pedigrees of rhesus macaque **(A)**, long-tailed macaque **(B)**, and Hamadryas baboon **(C)** families selected for *LILRB1* and *LILRB2* characterization, and a human family **(D)** for *LILRB1* characterization. In these pedigrees, males are represented by squares and females by ovals. For the Indian rhesus macaque, one male appears multiple times in the pedigree and is indicated by a square with a light-gray background. Similarly, for the long-tailed macaque, one female appears in multiple pedigrees and is represented by an oval with a light-gray background. In two Chinese rhesus macaque families, the mother’s identity is unknown, which is denoted as “not determined” (N.D.). In cases where the sex of the offspring is unknown, only the animal’s ID is provided. On two occasions, the segregation of *LILRB1* alleles could not be confirmed (**Supplementary Table 1**); in these instances, the specific animal IDs are underlined. J10006, J16012, J15021, J16019.

Representative image of an agarose gel with *LILRB1* amplicons size selected for downstream sequencing analysis. The FastRuler Middle Range DNA Ladder (L) of Thermo Fisher Scientific was used for size selection, with the length of the five purified DNA fragment illustrate the range of the selected products. Results on *LILRB1* characterization for J10006 (lane 2), J16012 (lane 3), J15021 (lane 4), and J16019 (lane 5) are depicted in **Supplementary Tables 1** and **9B** (animal IDs highlighted in red). The blanco (no-template control, lane 6) demonstrates the absence of DNA contamination.

Supplementary Figure 3 Phylogenetic analysis of *LILRB1* and *LILRB2* alleles from rhesus macaque (*Mamu*, blue), long-tailed macaque (*Mafa*, black), and Hamadryas baboon (*Paha*, green). The coding DNA sequences (CDS) of all *LILRB1* and *LILRB2* alleles identified at gDNA level, together with seven alleles detected only at the transcription level (**Supplementary Table 5**), were subjected to phylogenetic analysis. The Neighbor-joining tree was constructed using the Nei-Gojobori (Jukes-Cantor) method for evolutionary distances. Human *LILRB1* and *LILRB2* sequences (IDs shown in red and orange, respectively) were included in the analysis for comparison, and source information is provided in **Supplementary Table 4**. Relevant bootstrap values, based on 1,000 replicates, are indicated.

Supplementary Figure 4 Selected regions of the sequence alignments for *LILRB1* and *LILRB2* alleles, highlighting segments containing insertions and deletions. Human *LILRB* alleles (labeled Hosa, HM, AF283984, and AF283985) are shown at the top, followed by alleles from rhesus (Mamu) and long-tailed (Mafa) macaques, and Hamadryas baboon (Paha). “del.” denotes a deletion, “bp” indicates base pair, and “pos.” refers to position.

Supplementary Table 1 *LILRB1* and *LILRB2* allelic variation characterized in the rhesus macaque, long-tailed macaque and Hamadryas baboon animals. The data are presented by family (see also **Supplementary Figure 1**) and include allele characterization at both the cDNA and gDNA levels. Unrelated individuals are also included in the analyses for all species. If an allele could not be identified, it is denoted as “not determined” (N.D.). The underlined rhesus and long-tailed macaque IDs indicate the individuals for whom *LILRB1* characterization at the gDNA level was performed using Nanopore sequencing. An “L” is included in the allele name when only Nanopore sequencing was conducted for the corresponding animal, as it was unable to discriminate allelic variation. ^#^Occasions in which the segregation of a *LILRB1* allele could not be confirmed (see also **Supplementary Figure 1**), with the alleles involved underlined. In two Chinese rhesus families, alleles are printed in red to indicate that an intron mutation occurred when inherited from parent to child. As a result, these alleles received a different designation. Where known, males are depicted with a light-blue background, and females with a pink background. Animal IDs highlighted in red identify the animals whose *LILRB1* amplicons are depicted in **Supplementary Figure 2**.

Supplementary Table 2 Overview of primers designed for the amplification of *LILRB1* and *LILRB2* in rhesus (*Mamu*) and long-tailed macaque (*Mafa*) and Hamadryas baboon (*Paha*) at the transcription (cDNA) and genomic DNA (gDNA) level. The abbreviations Fw stands for forward, Rv for reverse. The location, the different annealing temperatures applied for each primer set and the expected product size (base pair, bp) amplified by the primers is indicated. The primers designed for *LILRB1* amplification in human (*Hosa*) samples are also included.

Supplementary Table 3 Information on the PacBio runs conducted to characterize allelic variation in *LILRB1* and *LILRB2* at both the transcription (cDNA) and genomic DNA (gDNA) levels. For the three species analyzed, the platform used, total number (#) of reads retrieved, number of samples, and the average number of reads per sample are provided. *Samples indicated for this sequel run were pooled together with other type of samples. The sequel I run, highlighted with a gray background, contained only four samples to ensure a higher number of reads per sample.

Supplementary Table 4 Overview of published, directly submitted and reference or predicted reference sequences included in the *LILRB1* and *LILRB2* reference library and phylogenetic analysis. ^The long-tailed macaque *LILRB2*, LOC102120967, is assigned as *Mafa-LILRB1* (5). #LOC102143922, LOC101022334, and LOC116271753, are identified as *Mafa-LILRB2*, *Paan-LILRB1*, and *Paan-LILRB2*, respectively, based on comparative analyses with the genomic organizations published by Storm et al., 2021.

Supplementary Table 5 Meta-data associated with the *LILRB1* and *LILRB2* alleles identified in rhesus and long-tailed macaques, and Hamadryas baboons. Bolded allele designations refer to alleles identified only at the transcriptional level. ENA-EMBL accession numbers for the alleles are provided. The number (#) of individuals in which each allele is detected is indicated (numbers extracted from **Supplementary Table 1**). If an allele is detected in a single individual, it is confirmed by an independent second PCR and an independent sequencing run (indicated as 2xPCR). For rhesus macaque alleles, the population in which the allele is detected (India, Burma, and China) is specified. A 2-field allele name is provided when alleles are detected at the transcriptional level. The suffix Sp indicates that the allele identified is an alternative splice variant. ^$^Alleles lacking exon 15 at the transcription level. ^#^Alleles lacking exon 9 at the transcription level. ^@^*Mamu-LILRB2*005:01:01:02* is identified in one individual with 56 reads from a single PCR, and further confirmation is required. ^^^*Mamu-LILRB2*006:01:01:02* and **006:01:01:03*, and *Mafa-LILRB2*004:02:01:01*, **004:04:01:01*, **008:01:01:01*, and **010:01:01:01* are identified in single individuals at both the cDNA and gDNA levels, with read numbers ranging from 51 to 1031 base pairs, confirming their coding sequences. However, intron sequences for these alleles require further confirmation. ^&^*Paha-LILRB2*013:02Sp* and **014:02* are identified in single individuals with read numbers ranging from 120 to 535 base pairs and require further confirmation. For each allele, the two nucleotides forming the splice site between exon 15-intron 15 (156 bp) are indicated. In addition, for the *LILRB1* alleles the two nucleotides forming the alternative splice site between exon 15-intron15 (126 bp) are provided. “N.D.” means not determined.

Supplementary Table 6 Numbers of synonymous (S) and non-synonymous (NS) mutations detected in *LILRB1* and *LILRB2* alleles from the studied rhesus macaque, long-tailed macaque and Hamadryas baboon populations. For comparison, the numbers on S and NS mutations detected in human *LILRB1* and *LILRB2* alleles are included. These human alleles correspond to those listed in **Supplementary Table 4**. The number (n) of macaque and baboon alleles included in the analysis are indicated. The length of each exon (in base pairs, bp) is provided and used to normalize the data. NA indicates “not applicable” and is used when the corresponding part of the sequence was not characterized or when the exon in question was not detected.

Supplementary Table 7 Polymorphic amino acid positions based on the deduced amino acid sequences of the rhesus (*Mamu*) and long-tailed (*Mafa*) macaque and Hamadryas baboon (*Paha*) LILRB1 (A) and LILRB2 (B) alleles for the D1 and D2 domain structures. The consensus sequences of human (*Hosa*) LILRB1 and LILRB2 are shown at the top and used as references. Identity with the reference is indicated by a dash (“-”), amino acid substitutions are represented by the standard *one-letter* code, and a deletion is indicated by an “x”. When polymorphisms are present within the *Hosa-*LILRB1 and *Hosa-*LILRB2 sequences, they are indicated using a slash between the relevant *one-letter* codes. The first row lists the allele names. When multiple alleles of a lineage share the same amino acid sequence, the digits identifying those alleles are listed next the first allele name, separated by a comma. The D1 domain is from position 25-121, the D2 domain from 122-221. For the rhesus macaque alleles, the geographic origin of the population in which they were detected is indicated. Position 72 and 103 in the human LILRB1 D1 domain, as discussed by Liu et al. (53), correspond to positions 73 and 104 in the alignment presented here, due to a insertion/deletion present at position 28.

Supplementary Table 8 Overview of the total number of human (*HLA*), rhesus macaque (*Mamu*), long-tailed macaque (*Mafa*), and Hamadryas baboon (*Paha*) MHC class I sequences and complete exon 4 sequences used for alpha 3 domain analysis **(A)** and the polymorphisms detected at different alpha 3 domain positions in the macaque and baboon species **(B)**. **(A)** The data were obtained from IPD-IMGT/HLA database release 3.60 and IPD-MHC NHP release 3.14.0.0. ^For the Hamadryas baboon, additional data from a manuscript currently in preparation are included. Sequences containing a premature stop codon were excluded from further analysis in **Supplementary Table 8B** and **Figure 6**. **(B)** Amino acid (Aa) positions discussed in the main text are highlighted with distinct background coloring. Identity with the consensus is indicated by a dash (“-”), amino acid substitutions are represented by the standard *one-letter* code, and a deletion is indicated by an asterisk (“*”). Polymorphisms within the sequences are denoted by a slash separating the relevant *one-letter* codes, with the number of sequences containing each polymorphism given in parentheses for positions included in **Figure 6**, as well as for other positions when the polymorphism is represented by only a small number of sequences.

Supplementary Table 9 Pivot tables showing the typing results of the rhesus macaque (*Mamu*) and long-tailed macaque (*Mafa*) *LILRB1* (A-B) and *LILRB2* (C-D) PacBio cDNA amplicons. Column A lists the different *LILRB1* or *LILRB2* variants. The animal IDs corresponding to each set of results are shown at the top of each column. Within each column, the distribution of reads retrieved per amplicon is indicated and corresponds to the allele or splice-variant designations listed in Column A. Some animal IDs appear two or three times, indicating that the same animal was sequenced in duplicate or triplicate. Animal IDs highlighted in red (**Supplementary Table 9B**) identify the animals whose *LILRB1* amplicons are depicted in **Supplementary Figure 2**.

Supplementary Table 10 Overview of alternative splicing events observed in the *LILRB1* (A) and *LILRB2* (B) transcriptomes of human (*Hosa*), rhesus macaque (*Mamu*), long-tailed macaque (*Mafa*), and Hamadryas baboon (*Paha*). Only splicing events detected in at least two individuals/animals and across two or more species are considered. The numbers indicate the distinct *LILRB1* and *LILRB2* alleles in which each alternative splicing event was identified. Different types of alternative splicing events within an array are separated by a period. Within each array, “bp” denotes base pair, “del” deletion, “ins” insertion, “ex” exon, and “in” intron. The mechanism of each splicing event is indicated in brackets. Alternative splicing events shown in **Figure 8** are indicated in bold.
