# Supplementary material for: LILRB1 and LILRB2 genomics and transcriptomics in macaque and baboon species: polymorphism, diversification, and extensive alternative splicing
Source: Front Immunol. 2026 Jan 9;16:1706720. doi: 10.3389/fimmu.2025.1706720 (PMC12827074; doi:10.3389/fimmu.2025.1706720)

figS01a

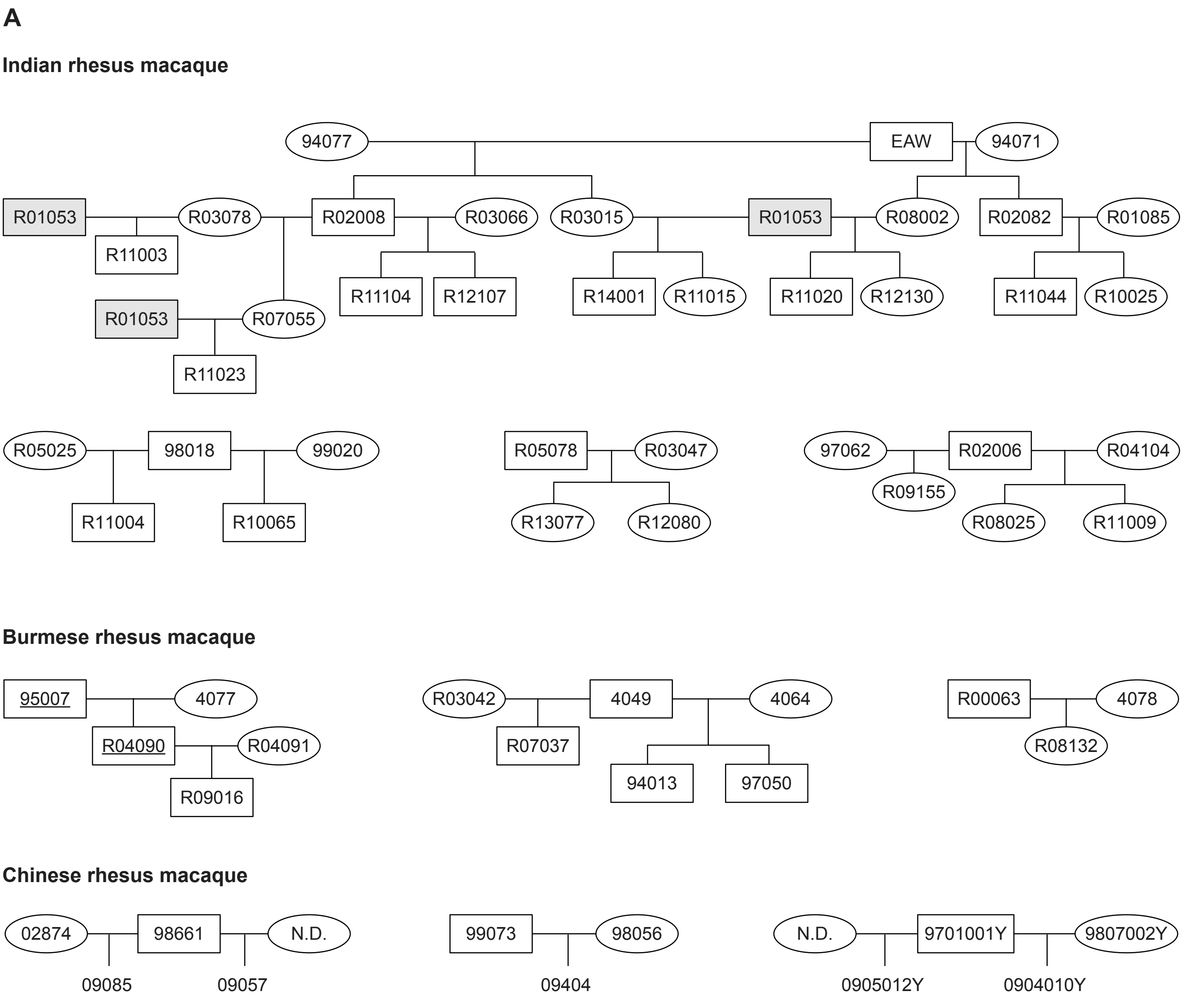
A
Indian rhesus macaque
94077
EAW
94071
R01053
R11003
R03078
R02008
R03066
R03015
R01053
R08002
R02082
R01085
R11104
R12107
R14001
R11015
R11020
R12130
R11044
R10025
R01053
R07055
R11023
R05025
98018
99020
R11004
R10065
R05078
R03047
R13077
R12080
97062
R02006
R04104
R09155
R08025
R11009
Burmese rhesus macaque
95007
4077
R04090
R04091
R09016
R03042
4049
4064
R07037
94013
97050
R00063
4078
R08132
Chinese rhesus macaque
02874
98661
N.D.
09085
09057
99073
98056
09404
N.D.
9701001Y
9807002Y
0905012Y
0904010Y

figS01b

B

Long-tailed macaque

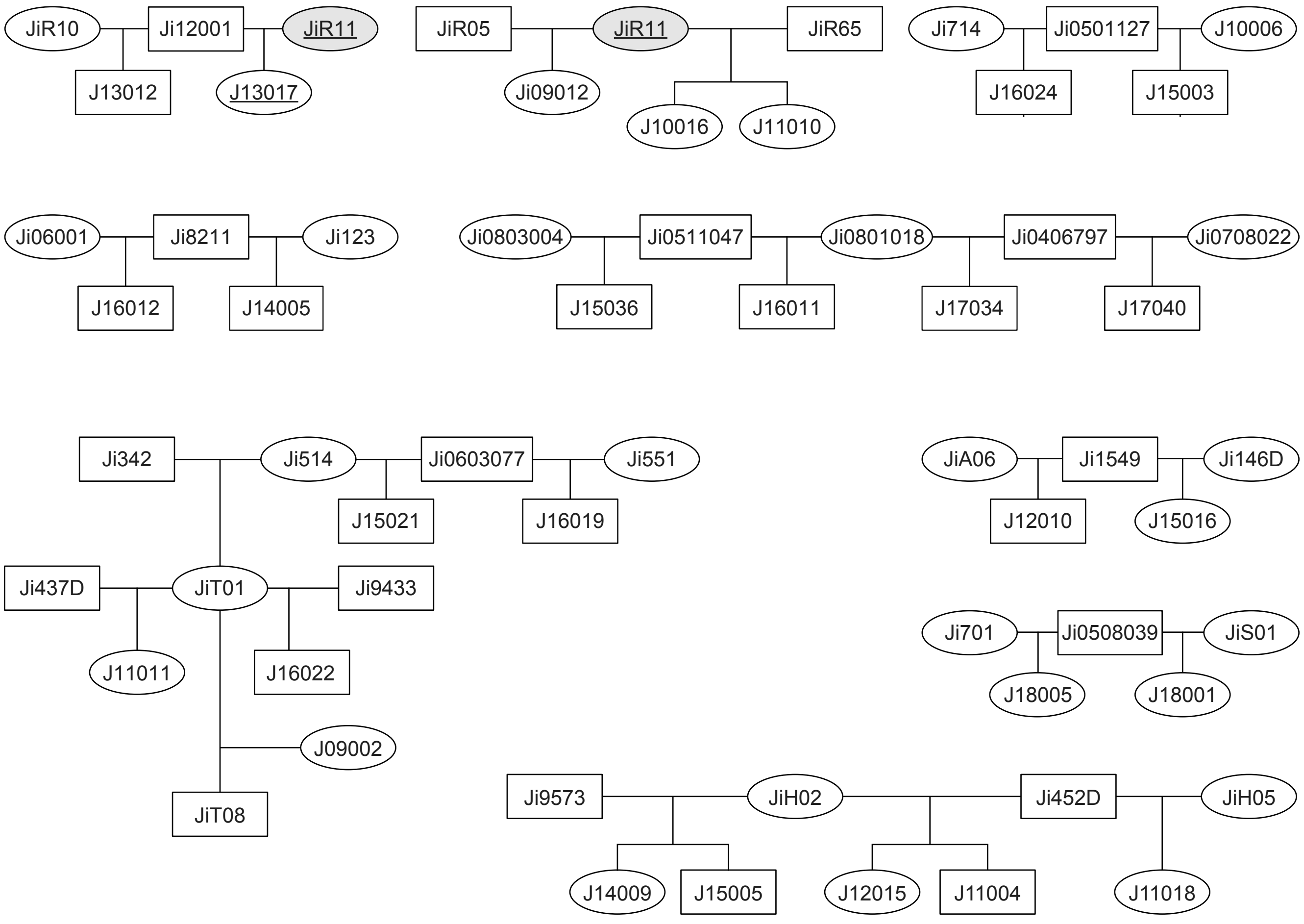

C

Hamadryas baboon

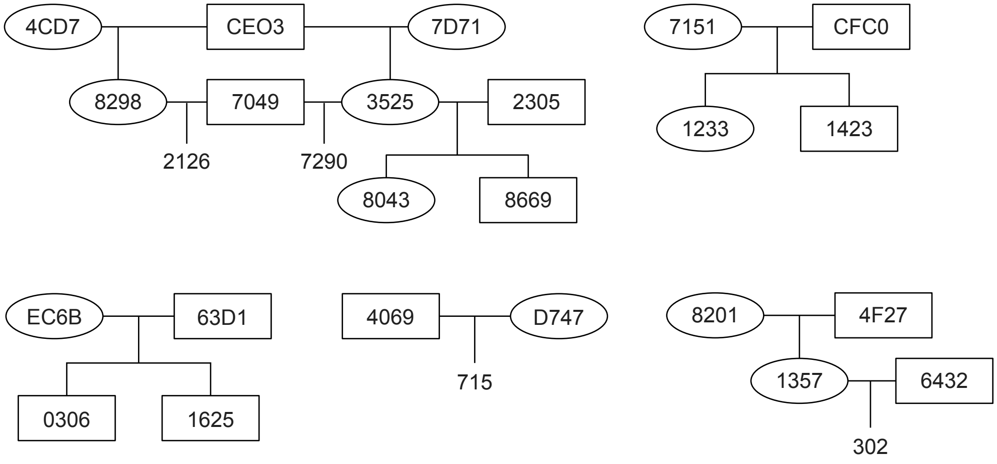

D

Human

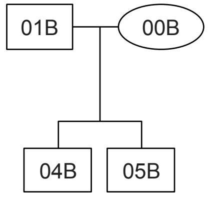

Supplement: Supplementary Figure 1 — Pedigrees of rhesus macaque (A), long-tailed macaque (B), and Hamadryas baboon (C) families selected for LILRB1 and LILRB2 characterization, and a human family (D) for LILRB1 characterization. In these pedigrees, males are represented by squares and females by ovals. For the Indian rhesus macaque, one male appears multiple times in the pedigree and is indicated by a square with a light-gray background. Similarly, for the long-tailed macaque, one female appears in multiple pedigrees and is represented by an oval with a light-gray background. In two Chinese rhesus macaque families, the mother’s identity is unknown, which is denoted as “not determined” (N.D.). In cases where the sex of the offspring is unknown, only the animal’s ID is provided. On two occasions, the segregation of LILRB1 alleles could not be confirmed (Supplementary Table 1); in these instances, the specific animal IDs are underlined. J10006, J16012, J15021, J16019. [file Image1.pdf]
