# Supplementary material for: LILRB1 and LILRB2 genomics and transcriptomics in macaque and baboon species: polymorphism, diversification, and extensive alternative splicing
Source: Front Immunol. 2026 Jan 9;16:1706720. doi: 10.3389/fimmu.2025.1706720 (PMC12827074; doi:10.3389/fimmu.2025.1706720)
Supplement: Supplementary file 2 — Representative image of an agarose gel with LILRB1 amplicons size selected for downstream sequencing analysis. The FastRuler Middle Range DNA Ladder (L) of Thermo Fisher Scientific was used for size selection, with the length of the five purified DNA fragment illustrate the range of the selected products. Results on LILRB1 characterization for J10006 (lane 2), J16012 (lane 3), J15021 (lane 4), and J16019 (lane 5) are depicted in Supplementary Tables 1 and 9B (animal IDs highlighted in red). The blanco (no-template control, lane 6) demonstrates the absence of DNA contamination. [file Image2.pdf]

FigS02

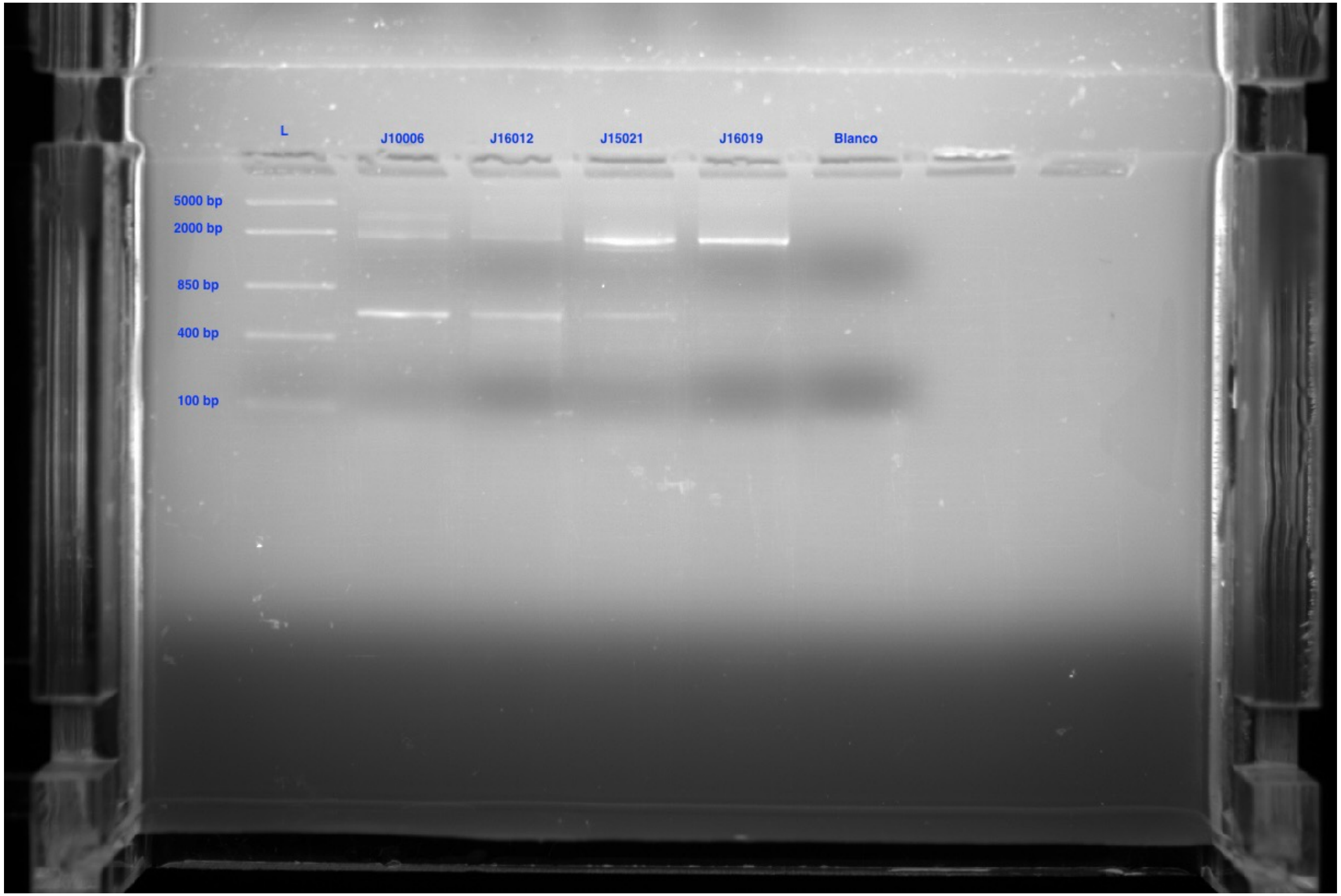
L
J10006
J16012
J15021
J16019
Blanco
5000 bp
2000 bp
850 bp
400 bp
100 bp

Filename: 2025-12-03 - 10.42.15.HSD5000

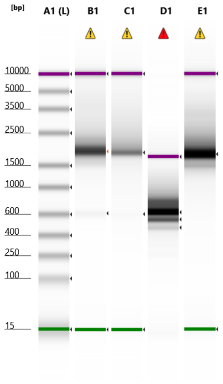

Default image (Contrast 100%)

Sample Info

Well	Conc. [pg/µl]	Sample Description	Alert	Observations
A1	2430	Ladder		Ladder
B1	1980	J10006	⚠	Sample concentration outside recommended range
C1	1220	J16012	⚠	Sample concentration outside recommended range
D1	7150	J15021	▲	Marker(s) not detected; Sample concentration outside recommended range
E1	4230	J16019	⚠	Sample concentration outside recommended range

© Agilent Technologies, Inc. 2023

A1: Ladder

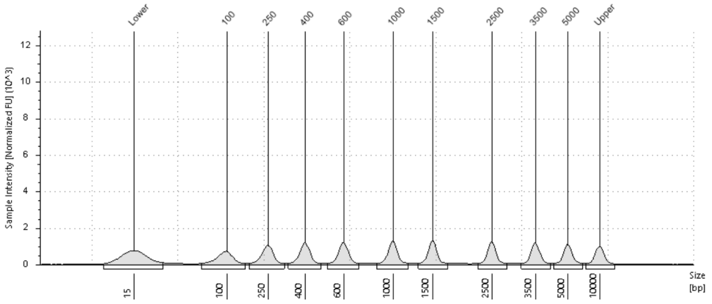

Sample Table

Well	Conc. [pg/µl]	Sample Description	Alert	Observations
A1	2430	Ladder		Ladder

Peak Table

Size [bp]	Calibrated Conc. [pg/µl]	Assigned Conc. [pg/µl]	Peak Molarity [pmol/l]	% Integrated Area	Peak Comment	Observations
15	369	-	37800	-		Lower Marker
100	263	-	4050	10.85		
250	284	-	1750	11.69		
400	290	-	1110	11.92		
600	283	-	726	11.65		
1000	284	-	436	11.68		
1500	276	-	283	11.34		
2500	258	-	159	10.61		
3500	253	-	111	10.42		
5000	239	-	73.5	9.84		
10000	180	180	27.7	-		Upper Marker

© Agilent Technologies, Inc. 2023

B1: J10006

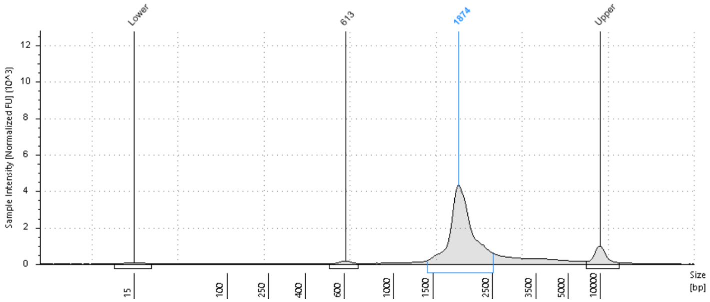

Sample Table

Well	Conc. [pg/µl]	Sample Description	Alert	Observations
B1	1980	J10006	⚠	Sample concentration outside recommended range

Peak Table

Size [bp]	Calibrated Conc. [pg/µl]	Assigned Conc. [pg/µl]	Peak Molarity [pmol/l]	% Integrated Area	Peak Comment	Observations
15	11.7	-	1200	-		Lower Marker
613	45.1	-	113	2.28		
1874	1930	-	1590	97.72		
10000	180	180	27.7	-		Upper Marker

© Agilent Technologies, Inc. 2023

C1: J16012

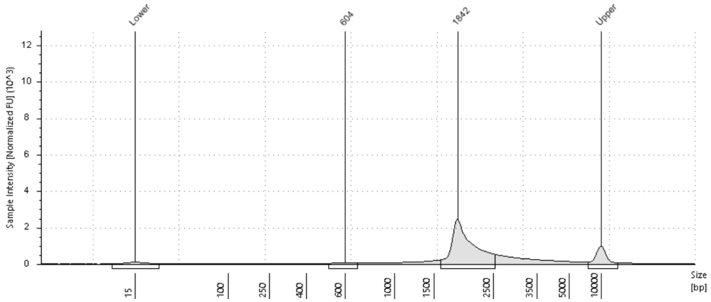

Sample Table

Well	Conc. [pg/µl]	Sample Description	Alert	Observations
C1	1220	J16012	⚠	Sample concentration outside recommended range

Peak Table

Size [bp]	Calibrated Conc. [pg/µl]	Assigned Conc. [pg/µl]	Peak Molarity [pmol/l]	% Integrated Area	Peak Comment	Observations
15	35.3	-	3620	-		Lower Marker
604	37.9	-	96.6	3.12		
1842	1180	-	984	96.88		
10000	180	180	27.7	-		Upper Marker

© Agilent Technologies, Inc. 2023

D1: J15021

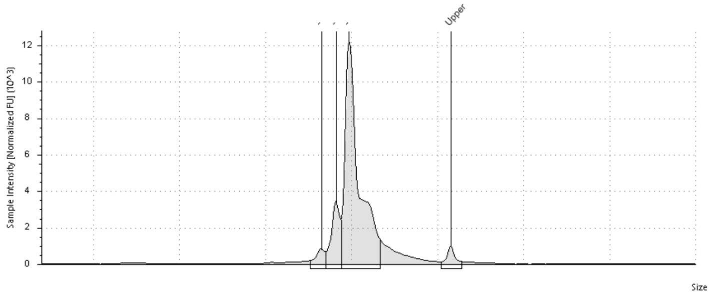

Sample Table

Well	Conc. [pg/µl]	Sample Description	Alert	Observations
D1	7150	J15021	▲	Marker(s) not detected; Sample concentration outside recommended range

Peak Table

Size [bp]	Calibrated Conc. [pg/µl]	Assigned Conc. [pg/µl]	Peak Molarity [pmol/l]	% Integrated Area	Peak Comment	Observations
-	270	-	-	3.78		
-	1020	-	-	14.26		
-	5860	-	-	81.96		
-	180	180	-	-		Upper Marker

Measurement repeated with a 1:1 dilution of the product. See next page

© Agilent Technologies, Inc. 2023

E1: J16019

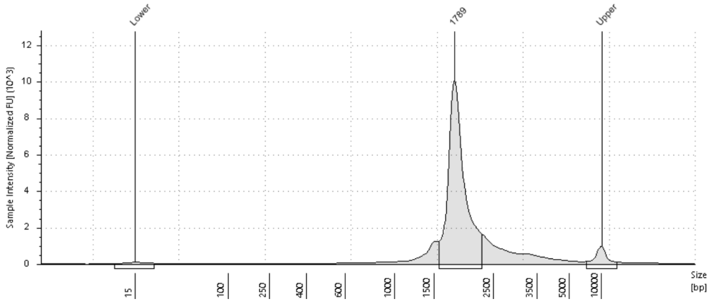

Sample Table

Well	Conc. [pg/µl]	Sample Description	Alert	Observations
E1	4230	J16019	⚠	Sample concentration outside recommended range

Peak Table

Size [bp]	Calibrated Conc. [pg/µl]	Assigned Conc. [pg/µl]	Peak Molarity [pmol/l]	% Integrated Area	Peak Comment	Observations
15	22.4	-	2290	-		Lower Marker
1789	4230	-	3640	100.00		
10000	180	180	27.7	-		Upper Marker

© Agilent Technologies, Inc. 2023

Filename: 2025-12-03 - 11.28.47.HSD5000

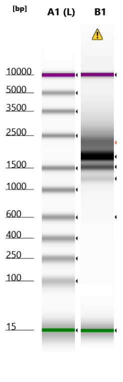

Default image (Contrast 100%)

Sample Info

Well	Conc. [pg/µl]	Sample Description	Alert	Observations
A1	1700	Ladder		Ladder
B1	3800	J15021	⚠	Sample concentration outside recommended range

© Agilent Technologies, Inc. 2023

B1: J15021

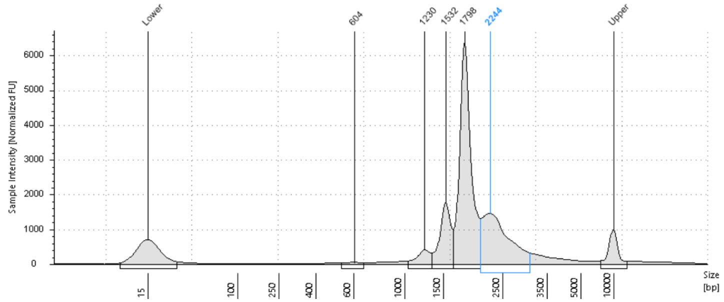

Sample Table

Well	Conc. [pg/µl]	Sample Description	Alert	Observations
B1	3800	J15021	⚠	Sample concentration outside recommended range

Peak Table

Size [bp]	Calibrated Conc. [pg/µl]	Assigned Conc. [pg/µl]	Peak Molarity [pmol/l]	% Integrated Area	Peak Comment	Observations
15	394	-	40400	-		Lower Marker
604	22.8	-	58.1	0.60		
1230	148	-	185	3.88		
1532	536	-	538	14.09		
1798	2020	-	1730	53.01		
2244	1080	-	741	28.43		
10000	180	180	27.7	-		Upper Marker

© Agilent Technologies, Inc. 2023
