# Supplementary figures and images for: LILRB1 and LILRB2 genomics and transcriptomics in macaque and baboon species: polymorphism, diversification, and extensive alternative splicing
Source: Front Immunol. 2026 Jan 9;16:1706720. doi: 10.3389/fimmu.2025.1706720 (PMC12827074; doi:10.3389/fimmu.2025.1706720)

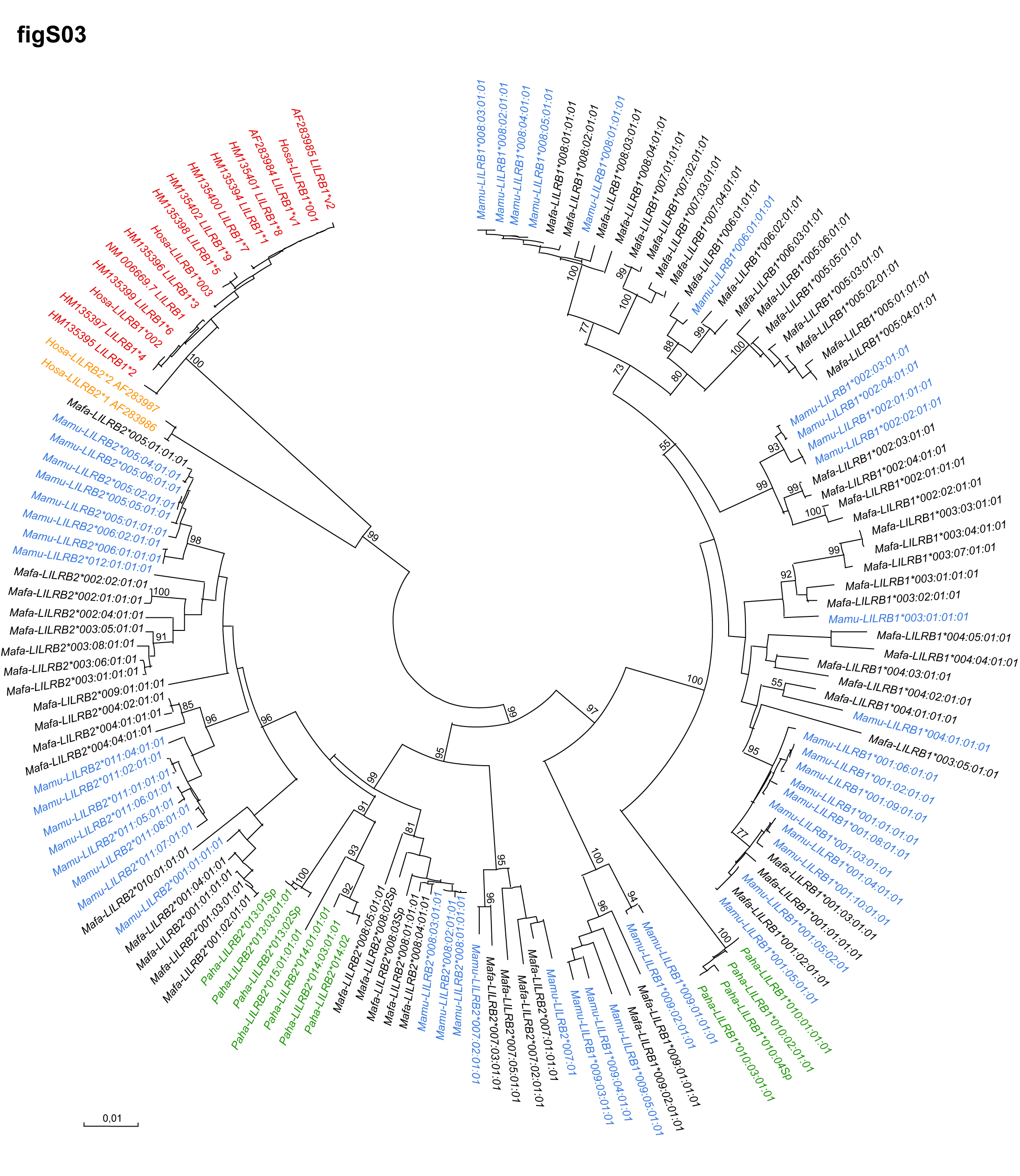

Supplement: Supplementary Figure 3 — Phylogenetic analysis of LILRB1 and LILRB2 alleles from rhesus macaque (Mamu, blue), long-tailed macaque (Mafa, black), and Hamadryas baboon (Paha, green). The coding DNA sequences (CDS) of all LILRB1 and LILRB2 alleles identified at gDNA level, together with seven alleles detected only at the transcription level (Supplementary Table 5), were subjected to phylogenetic analysis. The Neighbor-joining tree was constructed using the Nei-Gojobori (Jukes-Cantor) method for evolutionary distances. Human LILRB1 and LILRB2 sequences (IDs shown in red and orange, respectively) were included in the analysis for comparison, and source information is provided in Supplementary Table 4. Relevant bootstrap values, based on 1,000 replicates, are indicated. [file Image3.tif]

**figS04**

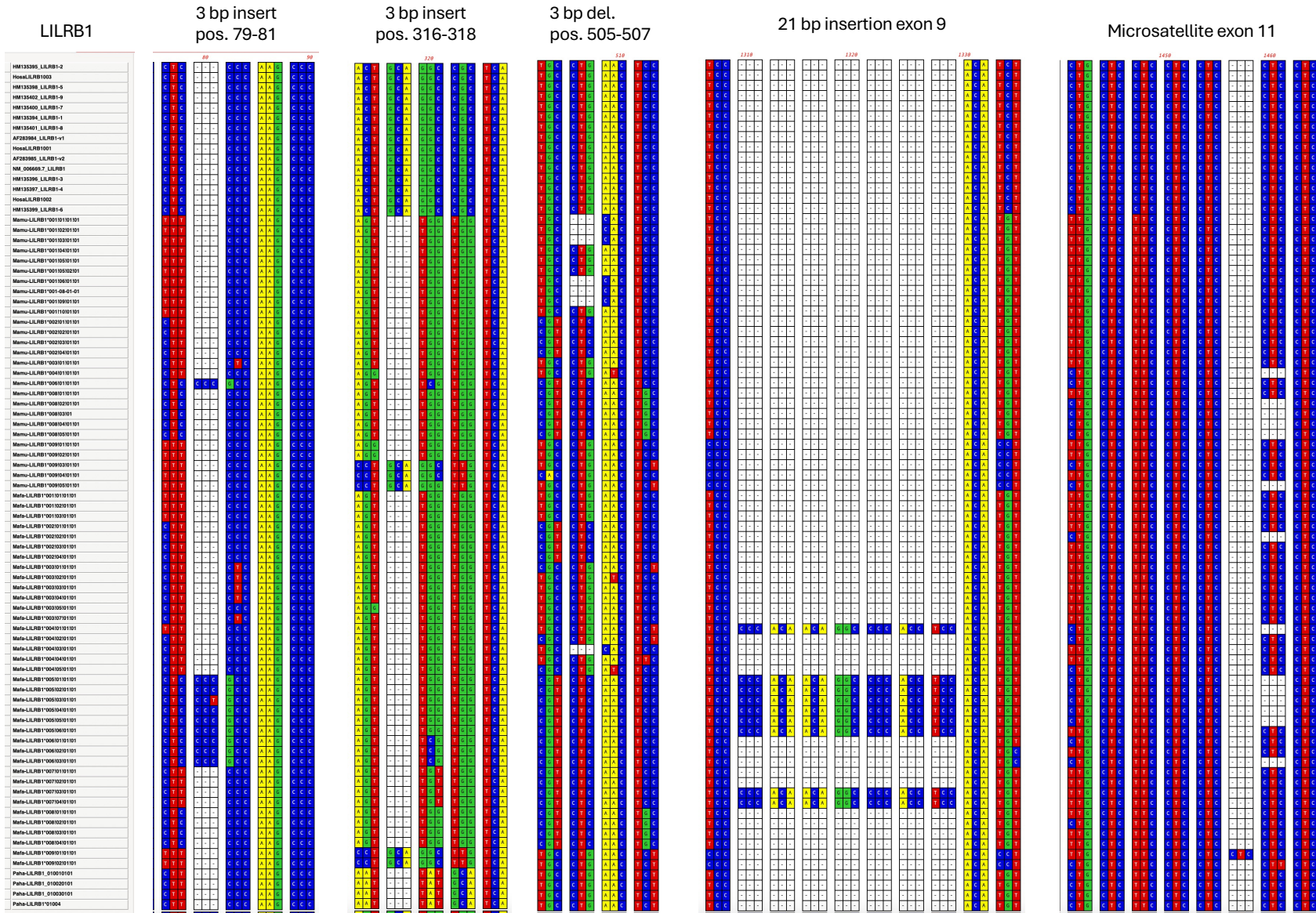

Supplement: Supplementary Figure 4 — Selected regions of the sequence alignments for LILRB1 and LILRB2 alleles, highlighting segments containing insertions and deletions. Human LILRB alleles (labeled Hosa, HM, AF283984, and AF283985) are shown at the top, followed by alleles from rhesus (Mamu) and long-tailed (Mafa) macaques, and Hamadryas baboon (Paha). “del.” denotes a deletion, “bp” indicates base pair, and “pos.” refers to position. [file Image4.pdf]
